# Transcriptome analysis reveals regulatory mechanisms of different drought-tolerant *Gleditsia sinensis* seedlings under drought stress

**DOI:** 10.1186/s12863-024-01216-y

**Published:** 2024-03-13

**Authors:** Fuhua Liu, Yang Zhao, Xiurong Wang, Biao Wang, Feng Xiao, Kequan He

**Affiliations:** 1 Institute for Forest Resources and Environment of Guizhou, College of Forestry, Guizhou University, Guiyang, 550025 Guizhou China; 2 Forestry Bureau of Qinglong, Qinglong, 561400 Guizhou China; 3 The State-Owned Forest Farm of Dushan County, Dushan, 558200 Guizhou China

**Keywords:** *G. sinensis*, Drought tolerant, Transcriptome analysis, Differentially expressed genes

## Abstract

**Background:**

*Gleditsia sinensis* is a significant tree species from both ecological and economic perspectives. However, its growth is hampered by temporary droughts during the seedling stage, thereby impeding the development of the *G. sinensis* industry. Drought stress and rehydration of semi-annual potted seedlings using an artificial simulated water control method. RNA sequencing (RNA-seq) analyses were conducted on leaves collected from highly resistant (HR) and highly susceptible (HS) seedling families at five different stages during the process of drought stress and rehydration to investigate their gene expression patterns.

**Results:**

The differentially expressed genes (DEGs) were predominantly enriched in pathways related to “chloroplast” (GO:0009507), “photosynthesis” (GO:0015979), “plant hormone signal transduction” (map04075), “flavonoid biosynthesis” (map00941), “stress response”, “response to reactive oxygen species (ROS)” (GO:0000302), “signal transduction” (GO:0007165) in *G. sinensis* HR and HS families exposed to mild and severe drought stress. Additionally, the pathways related to “plant hormone signal transduction” (map04075), and osmoregulation were also enriched. The difference in drought tolerance between the two families of *G. sinensis* may be associated with “transmembrane transporter activity” (GO:0022857), “stress response”, “hormones and signal transduction” (GO:0007165), “cutin, suberine and wax biosynthesis” (map00073), “ribosome” (map03010), “photosynthesis” (map00195), “sugar metabolism”, and others. An enrichment analysis of DEGs under severe drought stress suggests that the drought tolerance of both families may be related to “water-soluble vitamin metabolic process” (GO:0006767), “photosynthesis” (map00195), “plant hormone signal transduction” (map04075), “starch and sucrose metabolism” (map00500), and “galactose metabolism” (map00052). Osmoregulation-related genes such as *delta-1-pyrroline-5-carboxylate synthase* (*P5CS*), *Amino acid permease* (*AAP*), *Amino acid permease 2* (*AAP2*) and *Trehalose-phosphate synthase* (*TPS*), as well as the antioxidant enzyme *L-ascorbate peroxidase 6* (*APX6*), may be significant genes involved in drought tolerance in *G. sinensis*. Five genes were selected randomly to validate the RNA-seq results using quantitative real-time PCR (RT-qPCR) and they indicated that the transcriptome data were reliable.

**Conclusions:**

The study presents information on the molecular regulation of the drought tolerance mechanism in *G. sinensis* and provides a reference for further research on the molecular mechanisms involved in drought tolerance breeding of *G. sinensis*.

**Supplementary Information:**

The online version contains supplementary material available at 10.1186/s12863-024-01216-y.

## Background

Dramatic changes in the global climate and rainfall patterns have worsened the unequal distribution of water resources, resulting in drought conditions for numerous plant species [1]. Almost a third of the globe is in an arid or semi-arid state, and half of China's landmass is arid or semi-arid. Drought is a significant environmental factor that limits plant growth, and the decrease in crop yields due to drought surpasses all other environmental factors combined [2]. When plants experience drought stress, they produce substantial amounts of reactive oxygen species (ROS), which can harm the cell membranes and decrease light energy uptake. This can lead to irreversible damage to plant tissues or even plant death. The Guizhou terrain embodies a classic karst landscape, characterized by significant surface seepage and suboptimal soil moisture retention, which lead to slow vegetative growth and elevated seedling mortality during intermittent drought episodes. These factors impose significant constraints on the economic viability of the Guizhou region. While plants have developed various mechanisms to cope with harsh environments over extended periods of time, and drought tolerance is frequently enhanced through artificial selection and domestication, there is a relatively limited amount of research on the physiological and biochemical processes of drought tolerance. Thus, tackling the issue of drought tolerance at the molecular mechanistic level is difficult. When plants are subjected to drought stress, they combat the stress by initializing and controlling the expression patterns of pertinent drought-tolerant genes [3]. The complex mechanism of drought tolerance in plants is regulated simultaneously by multiple genes [4]. RNA-seq offers an effective means to investigate plant resistance and has evolved into a potent tool for elucidating drought stress and predicting gene function [5]. Furthermore, transcriptome analysis can illuminate the molecular mechanisms involved in specific biological processes [6]. Currently, RNA-seq studies have demonstrated the presence of drought-resistant genes in various plants, such as poplar [7], soybean [8] and maize [9]. These studies found that crops can enhance their drought tolerance by regulating metabolic pathways, including “abscisic acid (ABA)” and “Ca^2+^ signal pathways”, “jasmonic acid synthesis”, “glycolysis”, and “sugar metabolism”.

*Gleditsia sinensis* is a significant tree species for both ecology and economy, with wide distribution in China. It is one of the characteristic species for forestry development in Guizhou Province. The susceptibility of *G. sinensis* to drought is due to its specific geography, leading to a cyclical pattern of drought-rehydration-drought in its natural state. The seedling stage represents a crucial phase in plants' life cycle, whereby drought can exert a certain influence on the growth of *G. sinensis* seedlings. However, the variations in the impact deriving from different magnitudes of drought and rehydration across different families have not been thoroughly explored. Currently, studies centred around *G. sinensis* have emphasised seedling propagation methods [10], physiological seedling resilience [11, 12], and the morphology of pollen [13]. However, it fails to explore the molecular mechanisms of *G. sinensis*' adaptation to drought stress and the identification of its genes related to drought tolerance. This limitation impedes the study of drought tolerance mechanisms in *G. sinensis* from a molecular standpoint. In this study, an artificial simulation of water control was employed to treat semi-annual potted seedlings with drought stress and rehydration, and selected HR and HS families, and sampled seedlings of the two families in different treatment periods, and analyzed the gene expression in the leaves of *G. sinensis* seedlings when subjected to drought and rehydration by using RNA-seq technology, with the aim of discovering relevant drought resistant genes and exploring the molecular response mechanism of drought resistance of *G. sinensis* seedlings to provide theoretical basis for cultivation, production and popular application of *G. sinensis*. The aim is to explore the molecular response mechanism of drought resistance in *G. sinensis* seedlings, and provide a theoretical basis for the cultivation and application of *G. sinensis*, which will be helpful for the development and cultivation of drought resistant *G. sinensis* varieties.

## Materials and methods

### Plant material preparation

The test materials consisted of seeds collected from wild Gleditsia sinensis germplasm resources in Guizhou Province, as previously reported. The HS1 and HR1 families were identified in the collected seeds [14, 15]. The nursery soil underwent treatment with 0.1 percent potassium permanganate. The soil ratio used was humus: nutrient soil = 1:1. The pH of the potting soil was 7.53, with an average soil capacity of 1.25 g/cm^3^. The total nitrogen content was 10.84 g/kg, while the total phosphorus content was 0.97 g/kg, and the total potassium was 3.36 g/kg. Additionally, the organic matter content was measured to be 13.98 g/kg. Seedling pots measuring 24 cm in height and 20 cm in diameter were utilized to accommodate each seedling, with each pot being loaded with 80% of its volume in soil from the nursery. One seedling was planted per pot. Seedlings were selected for natural drought treatment after 5 months of growth and the test materials were well watered daily for 3 d prior to treatment, which was used as the soil saturated water content (soil water content of 30% to 35%). The soil moisture was measured using a soil moisture meter (Delta-T, UK) and averaged from three readings. Three drought gradients of mild drought (relative soil water content (percentage of maximum water holding capacity of the soil) of 55% ~ 60%) and severe drought (relative soil water content of 30% ~ 35%) were established, using 75% ~ 80% of relative soil water content as a control [16]. The natural drought time was determined by the water reduction to reach the drought interval, and the soil moisture content was determined to reach mild drought (7 d continuous drought) and severe drought (14 d continuous drought) using a soil moisture meter with the control (with relative soil water content of 75% ~ 80%) as 0 d. After 14 d of continuous drought, seedlings began to wilt and die. The rehydration test was carried out immediately after the drought test, during which the relative soil moisture content of the test seedlings was restored using a soil moisture meter. The collected leaves were snap frozen in liquid nitrogen and stored in a refrigerator at -80 ℃. Three biological replicates were established for each experimental treatment. Experimental and field studies on plants, including the collection of plant material and the conduct of plant trials, were conducted in accordance with relevant institutional, national and international guidelines and legislation, no competitive conflict of interest.

### RNA-Seq sequencing and data analysis

#### RNA extraction and transcriptome sequencing

Total RNA was extracted using RNAprep Pure Plant Kit (TIANGEN, Beijing, China), and the quality of RNA was assessed using 1% agarose gel electrophoresis, NanoDrop 2 000 (Thermo Fisher Scientific, USA) spectrophotometer detection and Agilent 5 300 (Agilent Technologies, USA). The RNA extraction quality and concentration of all samples were ≥ 1 ug total RNA, concentration ≥ 35 ng/μL, OD 260/280 ≥ 1.8, OD 260/230 ≥ 1.0, and bands were complete and clear with no apparent dispersion or tailing. A-T base pairing with ployA using magnetic beads with Oligo (dT) was used to isolate mRNA from total RNA. The fragments were randomly disrupted by adding fragmentation buffer and the double strand was synthesized by filtering the fragments. an 'A' base was added at the 3' end to connect the Y junction. The raw reads generated via Illumina sequencing were deposited in the NCBI SRA database (BioProject ID: PRJNA960694).

### Raw data pre-processing and unigenes function annotation

The raw reads were quality-controlled using fastp [17], the adapter sequences were removed, and low-quality reads were filtered out. The clean reads were then subjected to Trinity software for de novo assembly of the transcriptome. Trinity identified and reconstructed potential transcripts from the clean reads. The resulting assembled transcripts were subsequently utilized for further downstream analysis. To obtain a set of non-redundant sequences, the CD-HIT software was employed to select the longest coding sequences (CDS) as the representative unigenes [18, 19]. To obtain high-quality read data for sequence analysis, the raw reads containing adapter sequences and low-quality sequences were removed. After that, the clean reads were assembled into unigenes as the reference sequences using the Trinity (v2.4.0). The functions of unigenes were employed to determined using the Gene Ontology (GO, http://www.geneontology.org/), Protein family (Pfam, http://rfam.janelia.org/), Kyoto Encyclopedia of Genes and Genomes (KEGG, http://www.genome.jp/kegg/) [20], Clusters of Orthologous Groups of proteins (COG, http://www.ncbi.nlm.nih.gov/COG/), NCBI non-redundant protein sequence (NR, https://www.ncbi.nlm.nih.gov/public/), Swiss-Prot (Swiss-Prot protein database, http://www.gpmaw.com/html/swiss-prot.html).

### Weighted gene co-expression network analysis of differentially expressed genes

Clean data were aligned to reference data using Bowtie2 v2.3.5.1 software [21], and transcript level expression per detected expression was estimated in unit transcripts Per Kilobase of exon model per Million mapped reads (TPM) using RSEM v1.3.1 [22]. The number of genes identified was normalized using the DESeq2 R 4.3.1 software package [23]. DEGs screening threshold was set to *p*-value < 0.05 and |fold change|> 2. The results of the weighted correlation network analysis were determined using the WGCNA R 4.3.1 package [24], removing genes with TPM expression values below 1 in the sample. To identify enriched significant metabolic pathways and biological functions, we conducted KEGG pathway analysis and GO function enrichment using the clusterProfiler package [25], which screened for significant pathways with a threshold of corrected *p*-value < 0.05. The correlation between modules and physiological indicators of drought resistance in *G. sinensis* was calculated by applying the cor function, and the *p*-value was determined using the corPvalueStudent function. Heat mapping the correlations between modules and physiological indicators [26]. The target modules were selected for further study and essential genes were extracted.

### Quantitative real-time PCR validation

*EIF5A* was selested as the reference gene [27]. Five genes exhibiting differential expression greater than two fold were selected for analysis. Primers were designed using Primer 5.0 (Table 1). qRT-PCR was performed using a T100 Thermal CyclerP PCR instrument (BIO-RAD, USA). The reaction parameters for qRT-PCR were 95 °C/3 min; 95 °C/5 s, 60 °C/15 s, 40 cycles. The reaction system included 1.5 µl forward and reverse primers, 2.5 µl template cDNA, 12.5 µl 2 × SYBR Premix ExTaq, 8.5 µl ddH_2_O. The gene relative expression levels were calculated according to the 2^−∆∆Ct^ method [28]. Three technical replicates and three biological replicates were conducted.

**Table 1:** Primers sequence of qRT-PCR

Primers Name	Forward primer(5′-3′)	Reverse primer(5′-3′)
*TRINITY_DN2358_c0_g1*	ACATCATGACAACCTCAGCAGA	AGTAGCCAGTGTAGACACCAAT
*TRINITY_DN6754_c0_g1*	AGGCTTTGGCTTCTAAACTTCC	GCACCCCGTATACAATCTTGCT
*TRINITY_DN300_c0_g2*	TGCCAACATTCTGTCTTCCCAC	GCCACACACTTTTCCATCCACC
*TRINITY_DN14245_c0_g1*	GAAAAGCAGAAGAAGCAAGAGA	GTACCAAGGAAGTTTACAAGGG
*TRINITY_DN17273_c0_g1*	CCTTCAAAACCACAGTTTTCCC	TCCTCCAATTTCTCCATCCATC
*EIF5A*	CATGTGAATCGTACTGACTATC	GGTCATCCTTGGTGTTCC

### Statistical analysis

All statistical analyses were conducted using R v4.3.1 software [29]. To establish significance at a 0.05 probability level, the least significant difference (LSD) test was executed. The samples were analyzed using the hcluster function in cluster analysis.

## Results and analyses

### Analysis of RNA-seq and splicing results

After high-throughput sequencing, a total of 115.79G of data was obtained from 30 samples, with an average error rate of 0.02%, with an average Q20 > 98%, Q30 > 94%, and GC content > 44% (Table 2). A total of 1,588,719,568 high-quality clean reads were obtained, with an average of 52,957, 318 reads (> 8 Gb) for per samples, and the average percentage of uniquely mapped reads was 80.74% [30]. The 109,300 unigenes were annotated in six databases, included KEGG, COG, NR, Swiss-Prot, Pfam and GO (Table 3), of which 23,079 (21.21%) were annotated in KEGG, 48,564 (44.43%) in COG, 60,681 (55.52%) in NR and 37,628 (34.43%) in Swiss-Prot. The prediction of genes with encoded transcription factors (TFs) had the most unigenes belonging to the *MYB* superfamily with 198 unigenes, followed by the *AP2/ERF* family with 104 unigenes, and the number of unigenes was greater than 50 for the *C2C2* family, the *NAC* family, the *bHLH* family, the *WRKY* family, the *GRAS* family and the *bZIP* family.

**Table 2:** Transcriptome quality control results statistics

Sample	Raw reads Number	Raw bases	Clean reads	Clean bases	Error rate(%)	Q20	Q30	GC	Clean reads Mapped ratio
CHS 1	4.18E + 07	6.32E + 09	4.14E + 07	6.11E + 09	0.0255	97.92	93.7	43.44	79.42%
CHS 2	4.25E + 07	6.41E + 09	4.20E + 07	6.21E + 09	0.0261	97.67	93.09	43.43	78.38%
CHS 3	4.70E + 07	7.10E + 09	4.65E + 07	6.85E + 09	0.0258	97.81	93.4	43.32	78.14%
DHS 1	5.54E + 07	8.36E + 09	5.50E + 07	8.19E + 09	0.0258	97.80	93.32	44.69	78.90%
DHS 2	5.09E + 07	7.68E + 09	5.06E + 07	7.51E + 09	0.0254	97.97	93.75	44.81	79.90%
DHS 3	6.07E + 07	9.16E + 09	6.01E + 07	8.86E + 09	0.0263	97.61	92.93	44.92	80.14%
SHS 1	5.73E + 07	8.65E + 09	5.69E + 07	8.45E + 09	0.0256	97.87	93.54	44.19	78.42%
SHS 2	4.80E + 07	7.24E + 09	4.77E + 07	7.07E + 09	0.025	98.15	94.12	44.36	79.31%
SHS 3	4.95E + 07	7.48E + 09	4.92E + 07	7.26E + 09	0.0264	97.55	92.77	44.21	79.43%
rHS 1	6.34E + 07	9.58E + 09	6.30E + 07	9.24E + 09	0.0252	98.05	93.90	44.87	81.82%
rHS 2	4.64E + 07	7.00E + 09	4.60E + 07	6.70E + 09	0.0254	97.95	93.75	44.49	81.45%
rHS 3	6.55E + 07	9.89E + 09	6.51E + 07	9.58E + 09	0.0259	97.81	93.27	44.87	81.51%
RHS 1	6.28E + 07	9.49E + 09	6.24E + 07	9.22E + 09	0.0254	97.97	93.77	44.95	80.85%
RHS 2	5.46E + 07	8.25E + 09	5.42E + 07	7.92E + 09	0.0253	97.99	93.84	45.17	82.42%
RHS 3	5.55E + 07	8.38E + 09	5.52E + 07	8.10E + 09	0.0252	98.05	93.97	45.01	81.34%
CHR 1	4.86E + 07	7.33E + 09	4.82E + 07	7.11E + 09	0.0251	98.09	94.02	44.70	80.54%
CHR 2	5.28E + 07	7.97E + 09	5.23E + 07	7.71E + 09	0.0258	97.78	93.33	44.66	80.29%
CHR 3	5.47E + 07	8.27E + 09	5.43E + 07	8.01E + 09	0.0254	97.97	93.77	44.75	80.44%
DHR 1	5.37E + 07	8.11E + 09	5.34E + 07	7.90E + 09	0.0256	97.86	93.51	45.17	80.82%
DHR 2	5.17E + 07	7.81E + 09	5.13E + 07	7.50E + 09	0.0253	98.00	93.88	45.42	83.16%
DHR 3	5.26E + 07	7.94E + 09	5.23E + 07	7.75E + 09	0.025	98.14	94.13	45.01	80.16%
SHR 1	5.18E + 07	7.82E + 09	5.15E + 07	7.64E + 09	0.0257	97.83	93.37	44.60	80.72%
SHR 2	4.87E + 07	7.36E + 09	4.84E + 07	7.16E + 09	0.0262	97.65	92.98	44.61	80.52%
SHR 3	5.02E + 07	7.58E + 09	4.99E + 07	7.38E + 09	0.0254	97.98	93.77	44.66	81.55%
rHR 1	5.35E + 07	8.08E + 09	5.31E + 07	7.81E + 09	0.0257	97.82	93.42	45.02	81.84%
rHR 2	5.18E + 07	7.82E + 09	5.14E + 07	7.59E + 09	0.0257	97.83	93.4	44.83	81.99%
rHR 3	4.93E + 07	7.44E + 09	4.90E + 07	7.26E + 09	0.0254	97.98	93.77	44.71	81.35%
RHR 1	5.94E + 07	8.96E + 09	5.89E + 07	8.68E + 09	0.0251	98.08	94.03	44.95	82.34%
RHR 2	6.55E + 07	9.90E + 09	6.50E + 07	9.54E + 09	0.0253	98.01	93.89	44.96	82.52%
RHR 3	5.48E + 07	8.28E + 09	5.44E + 07	7.96E + 09	0.0254	97.96	93.78	44.92	82.62%

**Table 3:** General annotation list of gene funtion

Values	KEGG	COG	NR	Swiss-Prot	Pfam	GO	Overall
Number	23079	48,564	60,681	37,628	28,059	50,588	61,323
Percentage	21.12%	44.43%	55.52%	34.43%	25.67%	46.28%	56.11%

### DEGs analysis

**Fig. 1:**
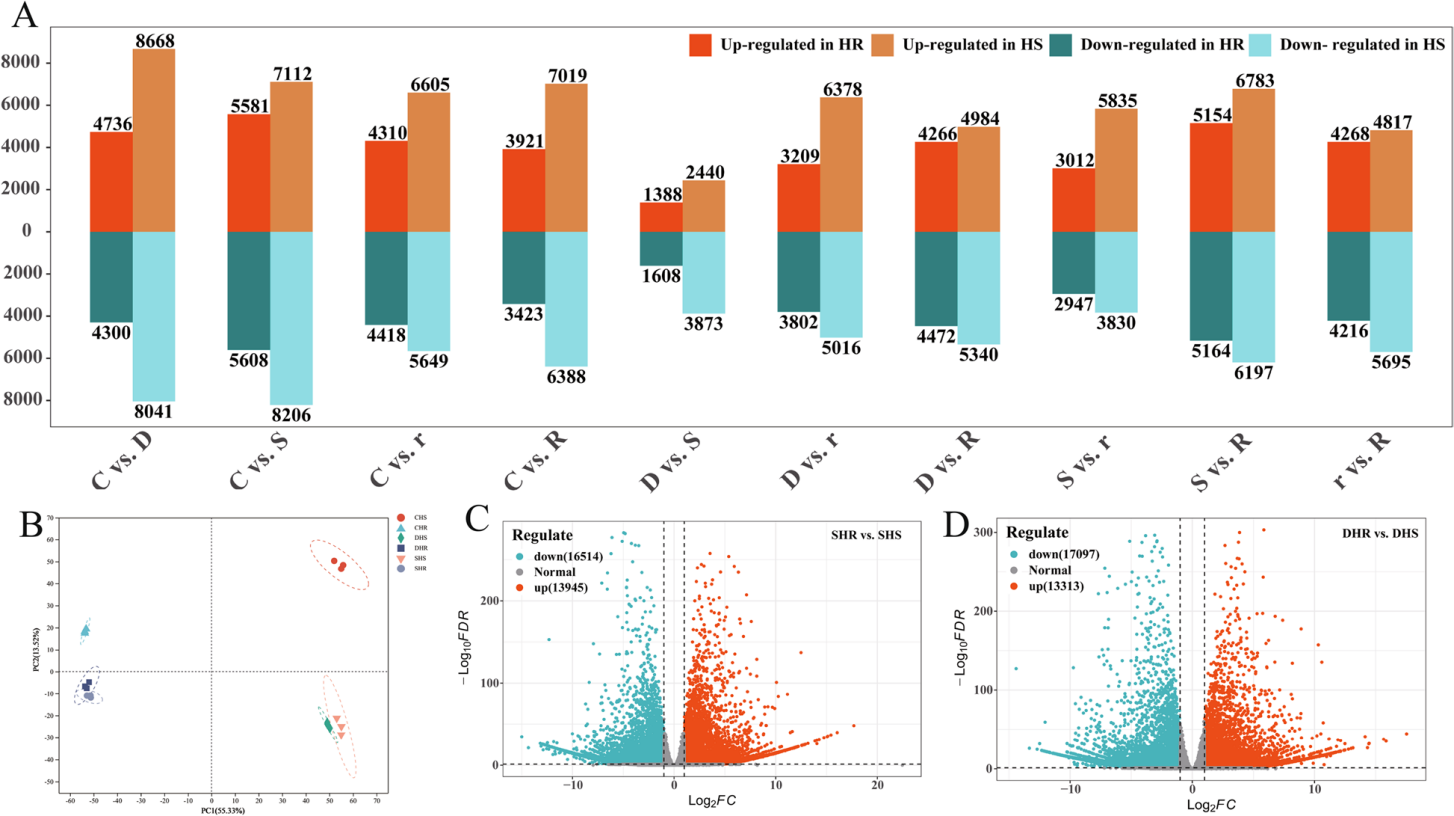
Differential expression of genes under different drought stresses and PCA

Principal component analysis (PCA) was used on the samples between treatments and it was found that the biological replicates clustered together, indicating specificity between each group of samples, demonstrating the reliability of the data (Fig. 1B). The distances between the same treatment in different families and between different treatments in the same family were greater, suggesting that *G. sinensis* seedlings respond to drought stress at different levels of drought through metabolite changes and thus to different levels of drought. Comparison of DEGs of *G. sinensis* families from HR and HS under successive drought and rehydration conditions (Fig. 1A). From the figure, the HS families had the highest number of DEGs compared to the mild drought control with 16,709 genes, of which 8,668 were up-regulated and 8,041 were down-regulated. For the HR families, the most DEGs were 11,189 in the severe drought group compared to the control group, including 5,581 up-regulated and 5,608 down-regulated genes. When comparing the periods together, the least number of genes were differentially expressed in the severe drought versus mild drought comparison, with a total of 2,996 DEGs in the HR families, of which 1,388 were up-regulated and 1,608 were down-regulated; and a total of 6,313 DEGs in the HS families, of which 2,440 genes were up-regulated and 3,873 genes were down-regulated. There were 30,459 DEGs in SHR vs SHS, of which 13,945 were up-regulated and 16,514 were down-regulated; and there were 30,410 DEGs in DHR vs DHS, of which 13,313 were up-regulated and 17,097 were down-regulated (Fig. 1C and Fig. 1D).

### GO and KEGG enrichment analysis of DEGs in *G. sinensis* in response to drought stress

**Fig. 2:**
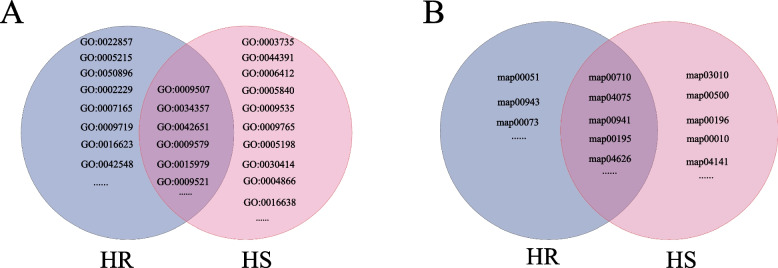
GO and KEGG enrichment analysis of DEGs in *G. sinensis in* response to drought stress. Note: **A**: GO enrichment analysis of DEGs, **B**: KEGG enrichment analysis of DEGs

GO and KEGG enrichment analyses of *G. sinensis* HR and HS families in response to mild and severe drought stress genes were performed and the results were shown below (Fig. 2). GO for DEGs in response to drought stress in both HR and HS families of *G. sinensis* included “chloroplast” (GO:0009507), “photosynthetic membrane” (GO:0034357), “thylakoid membrane” (GO:0042651), “thylakoid” (GO:0009579), “photosynthesis” (GO:0015979), “photosystem” (GO:0009521) and others. Comparison of GO enrichment between HR and HS families revealed that the GO of drought stress DEGs in response to drought stress in *G. sinensis* from families mainly included “transmembrane transporter activity” (GO:0022857), “transporter activity” (GO:0005215), “response to stimulus” (GO:0050896), “defense response” (GO:0006952), “signal transduction” (GO:0007165), “response to endogenous stimulus” (GO:0009719), “oxidoreductase activity” (GO:0016491), “acting on the aldehyde or oxo group of donors, oxygen as acceptor” (GO:0016623), “regulation of photosynthesis, light reaction” (GO:0042548) and others. GO of DEGs in response to drought stress in HS families of *G. sinensis* mainly included “structural component of ribosome” (GO:0005840), “ribosomal subunit” (GO:0044391), “translation”, “chloroplast” (GO:0009507), “thylakoid” (GO:0009579), “photosynthesis” (GO:0015979), “structural molecule activity” (GO:0005198), “peptidase inhibitor activity” (GO:0030414), “endopeptidase inhibitor activity” (GO:0004866), “oxidoreductase activity” (GO:0016491), “acting on the CH-NH_2_ group of donors” (GO:0016638) and others. The most significantly enriched pathways for DEGs in response to drought stress in both HR and HS families of *G. sinensis* included “metabolic pathways” and “biosynthesis of secondary metabolites”, “carbon fixation in photosynthetic organisms” (map00710), “plant hormone signal transduction” (map04075), “flavonoid biosynthesis” (map00941), “photosynthesis” (map00195), “plant-pathogen interaction” (map04626) and others. Comparison of the pathways of the HR and HS families revealed that the most significantly enriched pathways for DEGs in response to drought stress in the HR families of *G. sinensis* mainly included “fructose and mannose metabolism” (map00051), “cutin, suberine and wax biosynthesis” (map00073) and others. The most significantly enriched pathways for DEGs in response to drought stress in the HS families of *G. sinensis* mainly included “ribosome” (map03010), “starch and sucrose metabolism” (map00500), “photosynthesis-antenna proteins” (map00196), “glycolysis/ gluconeogenesis” (map00010), “protein processing in endoplasmic reticulum” (map04141) and others. Suggesting that the functions of DEGs in *G. sinensis* in response to drought stress were mostly related to “chloroplast” (GO:0009507), “thylakoid” (GO:0009579), “photosynthesis” (map00195), “metabolic pathways”, “plant hormone signal transduction” (map04075), “flavonoid biosynthesis” (map00941), “plant-pathogen interaction” (map04626) and others. The difference in drought tolerance between the two families of *G. sinensis* may be associated with “transmembrane transporter activity” (GO:0022857), “response to stimulus” (GO:0050896), “cutin, suberine and wax biosynthesis” (map00073), “translation”, “ribosome” (map03010), “photosynthesis” (map00195), “glucose metabolism” and others.

### Enrichment analysis of *G. sinensis* HS and HR families in response to severe drought stress DEGs

**Fig. 3:**
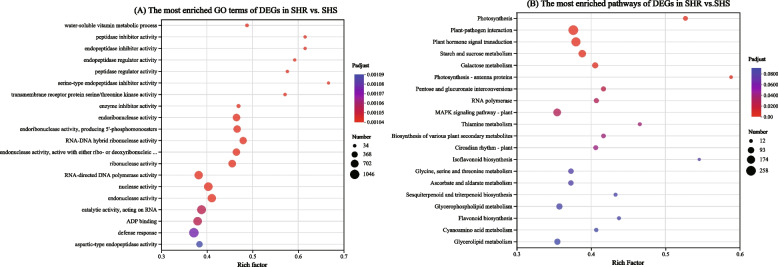
Enrichment analysis of HR families and HS families of *G. sinensis* in response to severe drought stress DEGs

To investigate the molecular functions of DEGs in response to severe drought in different drought-tolerant *G. sinensis* families, GO and KEGG enrichment analyses of HS and HR genes in *G. sinensis* under severe drought stress revealed (Fig. 3). The most significantly enriched GO included “water-soluble vitamin metabolic process” (GO:0006767), “peptidase inhibitor activity” (GO:0030414), “endopeptidase inhibitor activity” (GO:0004866), “endopeptidase regulator activity” (GO:0061135), “peptidase regulator activity” (GO:0061134), “serine-type endopeptidase inhibitor activity” (GO:0004867), “transmembrane receptor protein serine/threonine kinase activity” (GO:0004675) and “defense response” (GO:0006952), and others. The most significantly enriched “pathways included photosynthesis” (map00195), “plant-pathogen interaction” (map04626), “plant hormone signal transduction” (map04075), “starch and sucrose metabolism” (map00500), “galactose metabolism” (map00052), “photosynthesis-antenna proteins” (map00196), “pentose and glucuronate interconversions” (map00040), “RNA polymerase” (map03020), “MAPK signaling pathway-plant” (map04016) and others. Suggesting that the cause of the difference in drought tolerance between the two *G. sinensis* families may be related to “water-soluble vitamin metabolic process” (GO:0006767), “photosynthesis” (map00195), “plant hormone signal transduction” (map04075), “starch and sucrose metabolism” (map00500), “galactose metabolism” (map00052) and others.

### DEGs associated with osmoregulation and antioxidant enzyme activities under drought stress in *G. sinensis*

**Fig. 4:**
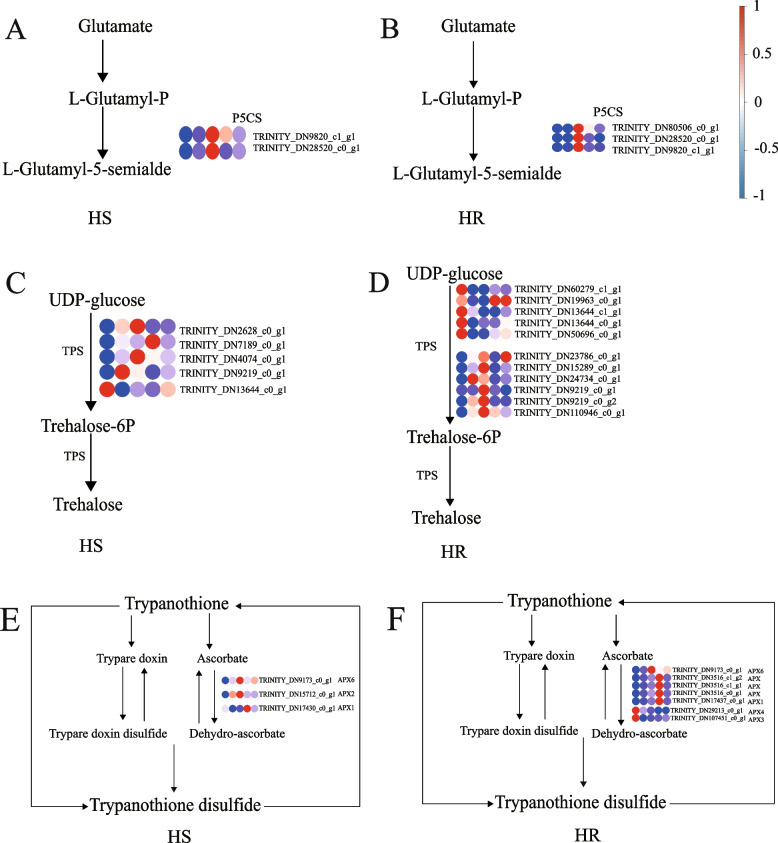
DEGs associated with arginine and *Pro* metabolism, starch and sucrose metabolism, and glutathione metabolism pathways in seedlings of *G. sinensis* under severe drought stress. Note: **A**, **B**: arginine and *Pro* metabolism; **C**, **D**: starch and sucrose metabolism; **E**, **F**: glutathione metabolism

To study the DEGs related to “arginine and proline (Pro) metabolism” (map00300), “starch and sucrose” (map00500) and “glutathione metabolism” (map00480) in the leaves of *G. sinensis* seedlings from HS and HR under drought stress, eighteen DEGs related to the synthesis of osmoregulatory substances and eight DEGs related to antioxidant enzyme activities were identified (Fig. 4). Under severe drought stress in *G. sinensis*, all four *P5CS* genes of Pro synthase were significantly up-regulated, promoting the conversion of glutamate to protein. *AAP* and *AAP2*, which were involved in Pro transport, were also significantly up-regulated (Table 4), suggesting that *P5CS*, *AAP* and *AAP2* play important roles in promoting Pro accumulation and were important osmoregulatory genes in drought tolerance in *G. sinensis*. *Sucrose synthase* (*SUS*) and *Alpha-amylase* (*AMY*) were significantly up-regulated under drought stress, increasing soluble sugar(SS) content, but *TPS* was significantly down-regulated under drought stress, with significant differences in the expression of 13 *TPS* genes, *TRINITY_DN60279_c1_g1*, *TRINITY_DN19963_c0_g1*, *TRINITY_DN13644_c1_g1*, *TRINITY_DN13644_c0_g1*, and *TRINITY_DN50696_c0_g1* were significantly down-regulated to inhibit starch and UDP-glucose synthesis of trehalose in sucrose metabolism, and the remaining eight genes were significantly up-regulated, indicating that the reduction of SS content in *TPS* under drought stress was mainly related to the reduction of SS synthesis. *TRINITY_DN13644_c1_g1* exhibited significant down-regulation in seedlings from both families and is likely a crucial gene in the regulation of starch and sucrose metabolism (map00500) in *G. sinensis*, which controls the reduction of SS content. Peroxidase (POD) genes (*TRINITY_DN70815_c0_g1*, *TRINITY_DN16226_c0_g1*) was significantly down-regulated under severe drought stress and ascorbate POD genes (*TRINITY_DN9173_c0_g1*) was significantly up-regulated in seedlings of both families. The expression of antioxidant enzymes was both up-regulated and down-regulated under drought stress in *G. sinensis* and did not show any consistency. The increased activity of POD in *G. sinensis* may be related to the significant up-regulation of *APX6*, which may be an important regulatory gene under drought stress in *G. sinensis*.

**Table 4:** Differential expression of osmoregulatory and antioxidant enzyme-related genes under drought stress in *G. sinensis*

Gene ID	Gene	HR			HS			Gene annotation
		C	D	S	C	D	S	
*TRINITY_DN9820 c1 g1*	*P5CS*	5.56	5.13	**143.30**	41.33	**74.06**	**385.53**	Delta-1-pyrroline-5-carboxylate synthase
*TRINITY_DN27120 c0 g1*	*AAP*	1.84	**4.05**	**67.99**	0.17	**14.55**	**92.66**	Amino acid permease
*TRINITY_DN566 c0 g2*	*AAP2*	1.14	1.46	**10.24**	2.06	0.88	**7.00**	Amino acid permease 2
*TRINITY_DN13644 c0 g1*	*TPS*	28.24	**6.67**	**6.92**	28.21	**8.99**	**14.15**	Trehalose-phosphate synthase
*TRINITY_DN3598 c0 g2*	*SUS*	0.08	**0.98**	**1.62**	0.37	**1.21**	0.61	Sucrose synthase
*TRINITY_DN9079 c0 g1*	*SPS*	60.06	**9.12**	**8.16**	35.68	**13.24**	**10.68**	Sucrose-phosphate synthase
*TRINITY_DN12386 c0 g1*	*HK*	5.09	5.95	8.06	3.76	**10.45**	**13.13**	Hexokinase-3
*TRINITY_DN16739 c0 g1*	*AMY*	0.32	**1.15**	**4.88**	2.66	4.20	**16.84**	Alpha-amylase
*TRINITY_DN1192 c0 g1*	*AMY*	6.04	**13.71**	12.33	26.22	27.98	**93.35**	Alpha-amylase
*TRINITY_DN70815 c0 g1*	*PER3*	1.74	**0.58**	**0.84**	0.72	0.25	**0.12**	Peroxidase 3
*TRINITY_DN28075 c0 g1*	*PER12*	0.19	0.55	0.16	0.39	**2.15**	**3.26**	Peroxidase 12
*TRINITY_DN120196 c0 g1*	*PER25*	2.36	**0.93**	**0.47**	0.70	0.95	0.31	Peroxidase 25
*TRINITY_DN9173 c0 g1*	*APX6*	3.94	6.04	**21.20**	15.12	**48.08**	**100.72**	L-ascorbate peroxidase 6
*TRINITY_DN16226 c0 g1*	*PER64*	0.32	**1.34**	**1.98**	0.43	0.58	**1.51**	Peroxidase 64
*TRINITY_DN110864 c0 g1*	*PER6*	1.92	**0.59**	**0.67**	0.02	0.08	0.02	Peroxidase 6
*TRINITY_DN3516 c0 g1*	*APX*	16.68	**38.59**	**95.81**	42.54	55.92	71.19	L-ascorbate peroxidase
*TRINITY_DN8953 c0 g2*	*PER5*	1.62	1.95	2.47	5.94	**1.98**	**0.95**	Peroxidase 5
*TRINITY_DN31762 c0 g1*	*PER47*	0.08	0.31	**0.61**	0.00	0.02	**1.96**	Peroxidase 47
*TRINITY_DN34397 c0 g1*	*PER55*	9.06	**4.48**	**2.84**	9.77	5.65	3.73	Peroxidase 55
*TRINITY_DN20779 c0 g1*	*PER45*	37.80	**13.64**	**19.17**	36.39	**5.73**	24.03	Peroxidase 45
*TRINITY_DN29794 c1 g1*	*CAT*	0.03	0.01	0.01	3.45	**0.06**	**0.04**	Peroxisomal catalase
*TRINITY_DN50320 c0 g1*	*SOD1*	0.03	0.00	0.12	1.71	**0.10**	**0.00**	Superoxide dismutase
*TRINITY_DN69713 c0 g4*	*SOD1*	0.00	0.09	0.03	1.26	**0.08**	**0.12**	Superoxide dismutase

Bold indicates differential expression compared to controls

### Weighted gene co-expression network analysis

Gene co-expression analysis is susceptible to the influence of abnormal samples, so it is important to exclude abnormal samples from the assay to improve the reliability of the results. The hierarchical clustering of samples was analyzed using the hclust function, and no sample outliers were found (Fig. S3). The soft thresholding power was set 7 (Fig. S3 C). Genes can be divided into 24 modules, among which the turquoise module has the highest number of genes, 6,672. followed by the blue module with 1,040 genes, and the darkgrey module has the lowest number of genes, only 116 (Fig. S3D).

**Fig. 5:**
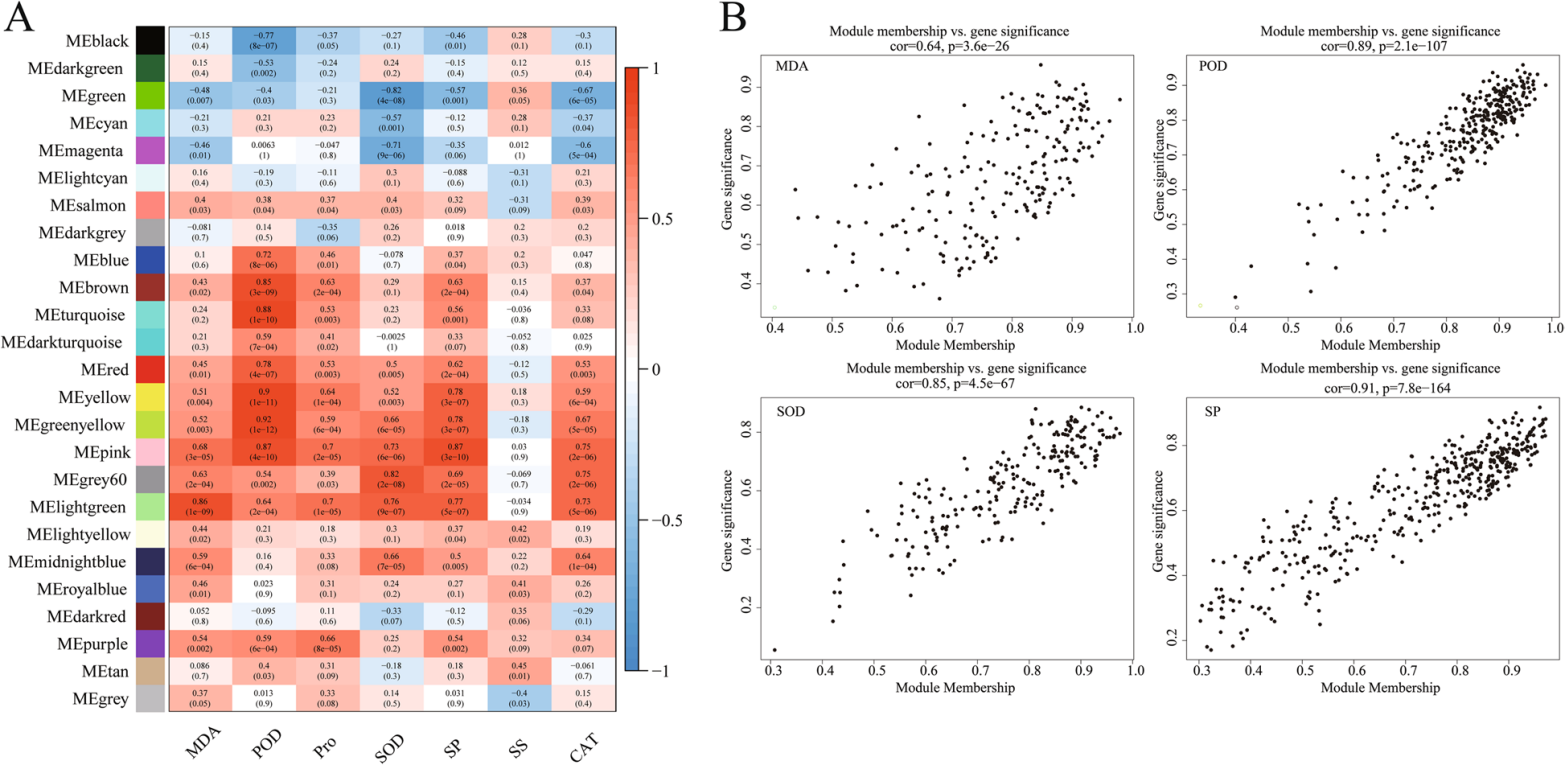
Gene module correlation and gene significance for drought traits in *G. sinensis*. Note: proline (Pro); soluble protein (SP); malondialdehyde (MDA); catalase (CAT); peroxidase (POD); superoxide dismutase (SOD); soluble sugars (SS)

The correlation between *G. sinensis* drought physiological indicators and each module was high, except for SS, where the red, yellow, pink, lightgreen and greenyellow modules showed good positive correlation with *G. sinensis* drought physiology, and the black and green modules showed mainly negative correlation with *G. sinensis* drought physiology; Among the seven physiological indices, POD had the maximum correlation coefficient with module, its correlation coefficient with greenyellow module was 0.92 and module was significant with gene (*r* = 0.89, *p* < 2.1e-107) (Fig. 5); Malondialdehyde (MDA) had the highest correlation with the lightgreen module at 0.86, and the correlation between gene and module was as high as the correlation between gene and trait (*r* = 0.64, *p* < 3.6e-26); The highest correlation was found between soluble protein (SP) and the pink module at 0.87, and the correlation between genes and modules was similar to that between genes and traits (*r* = 0.91, *p* < 7.8e-164); Superoxide dismutase (SOD) had the highest correlation with the grey60 module at 0.82, and the correlation between genes and modules was similar to that between genes and traits (*r* = 0.85, *p* < 4.5e-67), the genes within these modules may contain important genes related to drought resistance in *G. sinensis*. Twenty-four genes, including  *TRINITY_DN8319_c0_g2*, *TRINITY_DN58972_c0_g1* and *TRINITY_DN15470_c0_g1*, which may be related to drought resistance in *G. sinensis*, were screened by combining gene-module correlation (Table 5).

**Table 5:** Expression of drought resistance genes related to *G. sinensis*

Gene ID	HR					HS				
	C	D	S	r	R	C	D	S	r	R
*TRINITY_DN6910 c0 g1*	1.69	4.80	42.32	15.98	4.38	8.54	19.14	150.15	46.24	10.92
*TRINITY_DN5130 c0 g2*	4.82	4.30	88.74	27.89	11.05	8.61	26.05	93.70	56.62	11.05
*TRINITY_DN15470 c0 g1*	0.03	0.28	2.69	2.34	0.35	0.03	0.32	40.88	2.61	0.07
*TRINITY_DN10316 c0 g1*	1.16	2.02	6.67	2.68	1.32	0.56	4.19	13.33	10.16	4.47
*TRINITY_DN892 c0 g1*	1.34	2.33	3.77	2.34	2.19	4.14	5.11	11.58	6.62	3.47
*TRINITY_DN37386 c0 g1*	49.24	12.96	15.45	71.18	94.64	15.07	11.64	3.14	5.97	47.41
*TRINITY_DN8319 c0 g2*	0.29	2.34	3.59	1.06	0.87	2.80	9.39	17.13	11.31	2.78
*TRINITY_DN58972 c0 g1*	0.12	0.73	1.41	0.26	0.49	1.51	1.50	5.08	2.85	0.39
*TRINITY_DN117058 c0 g1*	0.94	2.00	5.41	6.09	1.08	3.88	14.42	36.67	17.89	0.92
*TRINITY_DN12190 c0 g1*	2.32	9.26	24.44	7.01	3.44	16.84	25.87	52.12	16.88	10.21
*TRINITY_DN9572 c0 g1*	997.73	833.08	533.85	558.76	923.90	448.01	535.23	114.07	206.87	475.51
*TRINITY_DN25838 c0 g1*	53.36	18.13	29.11	28.65	41.23	27.92	11.90	5.73	13.63	17.79
*TRINITY_DN48351 c0 g1*	0.85	5.11	87.99	7.56	0.70	6.94	61.38	277.83	115.06	45.87
*TRINITY_DN8873 c0 g1*	92.07	44.12	25.32	81.04	91.70	71.98	16.45	27.92	37.31	45.10
*TRINITY_DN1797 c0 g1*	23.08	180.06	313.56	135.65	113.23	63.10	377.12	283.22	96.84	108.66
*TRINITY_DN6093 c0 g1*	0.57	3.21	4.12	1.47	0.90	1.55	7.02	4.73	1.65	2.53
*TRINITY_DN12129 c0 g1*	8.44	16.92	32.93	17.61	18.07	10.56	34.94	40.93	23.54	24.00
*TRINITY_DN9812 c0 g1*	3.79	11.36	16.05	12.16	11.34	6.79	17.23	19.70	9.70	8.40
*TRINITY_DN28431 c0 g1*	39.22	30.91	14.21	77.29	41.97	2.37	3.60	1.01	14.92	14.97
*TRINITY_DN6826 c0 g1*	37.59	28.76	19.13	41.49	31.12	14.46	15.31	6.50	21.69	22.72
*TRINITY_DN18557 c0 g1*	5.38	14.00	19.22	9.57	9.40	21.65	35.72	100.78	20.90	18.84
*TRINITY_DN22800 c0 g1*	28.47	12.88	12.09	58.70	33.69	6.45	4.00	1.77	2.96	3.73
*TRINITY_DN22090 c0 g1*	5.25	4.94	1.47	10.29	4.71	0.78	0.57	0.02	1.82	3.74
*TRINITY_DN6998 c0 g1*	32.63	3.41	1.42	22.80	13.72	0.53	0.05	0.02	1.39	3.16

### qRT-PCR validation

To validate the accuracy of the RNA-seq data, qRT-PCR was performed for five genes randomly selected from the DEGs. The results showed that the expression of the five genes in the HR and HS families of *G. sinensis* was consistent with the expression patterns of the RNA-seq data (Fig. S4). A strong positive correlation (*R*^*2*^ = 0.96) was obtained through linear regression analysis (Fig. S5), suggesting that the transcriptome data was reliable.

## Discussion

Plants' responses to drought stress are intricate and entail various hormone signals, water regulation, antioxidant defence systems, protein synthesis and modifications, as well as gene expression regulations. Various plant species and varieties can display unique response mechanisms, as well as adopt diverse physiological and molecular strategies, to tackle drought stress. In this study, the molecular regulatory mechanisms of *G. sinensis* seedlings under drought stress were investigated using the HS and HR families. 4,053 genes were found to be co-differentially expressed in the HS and HR families during severe drought. Enrichment of DEGs under mild and severe drought stress for “chloroplast” (GO:0009507), “photosynthesis” (map00195), “plant hormone signal transduction” (map04075), “flavonoid biosynthesis” (map00941), “stress response”, “response to ROS” (GO:0000302), “signal transduction” (GO:0007165) and “osmoregulation pathways” facilitate *G. sinensis* respond to drought stress. The difference in drought tolerance between the two families of *G. sinensis* may be associated with “transmembrane transporter activity” (GO:0022857), “stress response”, “plant hormone signal transduction” (map04075), “cutin, suberine and wax biosynthesis” (map00073), “ribosome” (map03010), “photosynthesis” (map00195), “sugar metabolism”, *etc*. Pro is an important osmotic regulator in plants under drought stress and is associated with oxidative responses that stimulate ROS production [31]. In the present study, *P5CS*, *AAP* and *AAP2* genes involved in osmotic regulation were significantly up-regulated and *TPS* genes were significantly down-regulated, which, together with *APX6* and other genes involved in antioxidant enzymes, may be important genes involved in drought tolerance in *G. sinensis*. Overexpressed *P5CS* gene increases tolerance to drought and salt stress in transgenic rice, tobacco, sugarcane and wheat by increasing Pro levels [32, 33]. DEGs in *Ulmus pumila* 'Zhonghua Jinye' [34] seedlings under drought stress were mainly enriched in “cellular fractions involved in membrane protein complexes”, “photosynthetic membranes”, “cytoplasmic fractions” and “intracellular non-membrane organelles”. DEGs from *Dontostemon elegans* [35] were mainly enriched in functions related to the “cell wall”, whereas DEGs from *Solanum tuberosum* L [36] stem segments were mainly enriched in functions related to “redox processes”, “oxidoreductase activity” and “hormone response”, this differs from the results of the present study, which may be due to differences in stress mode or drought tolerance of different plant materials, or differences in gene composition, homeostatic regulation and response signal of different plants. Enrichment analysis of DEGs under severe drought stress revealed that the drought tolerance of seedlings of the two families may be differentially related to “water-soluble vitamin metabolic process” (GO:0006767), “photosynthesis” (map00195), “plant hormone signal transduction” (map04075), “starch and sucrose metabolism” (map00500), “galactose metabolism” (map00052), *etc*. Plants adapt starch and sucrose synthesis, catabolism and transport to drought conditions. Genes involved in the regulation of starch and sucrose metabolism may play an important role in drought stress [37]. The results of this study provided information for elucidating the drought resistance of *G. sinensis* and a basis for identifying candidate genes for drought resistance.

Different plant tissues feel the drought stress time is not the same time, the first tissue to feel the drought stress is mainly the root system of the plant in direct contact with the soil, so the plant is often subject to drought stress through a variety of signaling factors, the transmission of drought signals to the various tissues of the plant, so that the various tissues of the plant in response to drought. There are many signal factors in plants, of which plant hormones are important components, and ABA is a common signal factor among plant hormones. In sweet sorghum seedlings [38], genes encoding the ABA receptors *PYR/PYL*, *SnRK2* and a *PP2C* gene were down-regulated and four genes encoding *PP2C* and one encoding *ABF* were up-regulated for expression under drought stress. ABA binds to the PYR/PYLs/RCARs receptor proteins to form a complex that inhibits PP2C, allowing the activation and release of SnRK2, which in turn phosphorylates downstream TFs, ion channels, activating the ABA signal pathway and the stress response process [39]. A number of phytohormones have also been shown to be important signal factors during drought stress in plants and to play an important role in plant drought [40]. After plants are exposed to drought stress, the leaf blade receives drought signals that trigger a series of responses to drought stress in which the photosynthetic process is affected, causing O^2^ to accept electrons and become a highly O^2−^, which causes membrane lipid peroxidation, destroying the cell membrane and generating MDA [41]. Oxidoreductases are enriched in plants during drought, converting O^2−^ to water and reducing oxidative damage to the plant [42]. The enrichment of DEGs for oxidoreductase activity (GO:0016491) in this study indicates that the enrichment of oxidoreductase plays an important role in drought stress in *G. sinensis*, which is the same as in oilseed rape [43]. The drought co-expressed genes of *G. sinensis* revealed that “photosynthesis (map00195) and “amino acid metabolism” play important roles in the regulation of drought tolerance in *G. sinensis*. Amino acids and their derivatives were also found to be significant differential metabolites enriched during drought in a metabolomics study of *Platycodon grandiflorus* [44].

Gene expression is regulated by TFs that modulate abiotic stress responses and improve stress tolerance in plants [45]. In the sequencing results of this study, the *MYB* superfamily family was the most abundant, followed by the *AP2/ERF* family, the *C2C2* family, the *bHLH* family, the *WRKY* family, *etc*., and the TFs of these families have been shown to be associated with drought tolerance in plants in previous studies [46]. *MYB* TFs have a variety of biological functions, including plant secondary metabolism, hormones and environmental factors [47], and the combination of key genes such as *MYB2*, *MYB96*, *etc*., can regulate ABA dependent signal pathways and enhance drought tolerance in plants [48]. The transcriptome of *Salvia miltiorrhiza* [49] under drought stress was studied and it was found that the *AP2/ERF*, *bHLH*, *bZIP*, *WRKY* and *MYB* families were the most differentially expressed in roots, and *AP2/ERF* was up-regulated in roots and *HSF* was up-regulated in both leaves and roots, suggesting that the TFs of these families have an important role to regulate in the presence of drought.

In this study, WGCNA analysis was employed to screen 24 genes that may be associated with drought tolerance in *G. sinensis*. *TRINITY_DN3885_c0_g1* and *TRINITY_DN10316_c0_g1* were identified in the NR database as aspartic proteases which participate in both protein processing/degradation and response to adversity [50, 51], and were involved in regulating dehydrin synthesis. *TRINITY_DN8319_c0_g2* and *TRINITY_DN58972_c0_g1* were annotated to F-box proteins, which play important roles in regulating the degradation of aberrant proteins and enhancing the activity of antioxidant enzymes. Overexpression of *FOF2* results in increased ABA levels, increased ABA sensitivity in the stomatal closure zone, and reduced water loss, which improves drought tolerance in plants [52]. *TRINITY_DN15470_c0_g1* and *TRINITY_DN12190_c0_g1* annotated to thioredoxin, which catalyses the thiol-disulfide bond exchange, regulates intracellular redox and antioxidant enzyme systems, and enhances drought tolerance in plants [53, 54]. *TRINITY_DN117058_c0_g1* and *TRINITY_DN12129_c0_g1* were annotated to ubiquitin ligase E3, the SCF protein complex, which degrades negatively regulated proteins in the antiretroviral response during drought and induces drought stress, or causes F-box proteins originally involved in degradation to be under-expressed and positively regulated proteins not degraded, thereby inducing drought stress [55]. The remaining genes were annotated as heat shock proteins, water channel proteins, sulfate transporter proteins, and potassium transporter proteins, among others. Previous studies have shown that these genes were related to drought stress [56, 57], and whether these genes play a role in drought tolerance in *G. sinensis* requires further investigation.

The present study only looked at physiological and molecular responses, but not at the cellular and other levels, and anatomical and other studies could be followed up. The genes screened in this study need to be functionally verified to further investigate the mechanism of drought resistance in *G. sinensis*.

## Conclusions

DEGs under drought stress in HR and HS seedling families of *G. sinensis* were mainly enriched in “chloroplast” (GO:0009507), “photosynthesis” (map00195), “plant hormone signal transduction” (map04075), “flavonoid biosynthesis” (map00941), “stress response”, “response to ROS” (GO:0000302), “signal transduction” (GO:0007165), osmoregulation and other related pathways. The difference in drought tolerance between the two families of *G. sinensis* may be associated with “transmembrane transporter activity” (GO:0022857), “stress response”, “plant hormone signal transduction” (map04075), “cutin, suberine and wax biosynthesis” (map00073), “ribosome” (map03010), “photosynthesis” (map00195), “sugar metabolism”, and other factors. Enrichment analysis of DEGs under severe drought stress revealed that the two families differ in drought tolerance possibly related to “water-soluble vitamin metabolic process” (GO:0006767), “photosynthesis” (map00195), “plant hormone signal transduction” (map04075), “starch and sucrose metabolism” (map00500), and “galactose metabolism” (map00052). *P5CS*,* AAP, AAP2,* and *TPS*, which are involved in osmoregulation, along with *APX6*, an antioxidant enzyme, may play crucial roles in drought tolerance in *G. sinensis*.  A combination of gene-module correlation and gene-indicator correlation was used to screen 24 genes that may be associated with drought resistance in *G. sinensis*. The results of the study may provide a reference for elucidating the physiological and molecular mechanisms of *G. sinensis* seedlings against drought stress.

### Supplementary Information

**Supplementary Material 1. **

## Data Availability

The raw reads generated via Illumina sequencing were deposited in the NCBI SRA database (BioProject ID: PRJNA960694).

## Abbreviations

*AAP*: Amino acid permease
*AAP2*: Amino acid permease 2
ABA: Abscisic acid
*AMY*: Alpha-amylase
*APX6*: L-ascorbate peroxidase 6
CAT: Catalase
CDS: Coding sequences
COG: Clusters of Orthologous Groups of proteins
DEGs: Differentially expressed genes
*EIF5A*: Eukaryotic translation initiation factor 5A
GO: Gene Ontology
HR: Highly resistant
HS: Highly susceptible
KEGG: Kyoto Encyclopedia of Genes and Genomes
LSD: Least significant difference
MDA: Malondialdehyde
NR: NCBI non-redundant protein sequence
PCA: Principal component analysis
Pfam: Protein family
POD: Peroxidase
*P5CS*: 1-Pyrroline-5-carboxylate synthase
Pro: Proline
qRT-PCR: Quantitative real-time PCR
RNA-seq: RNA sequencing
ROS: Reactive oxygen species
SP: Soluble protein
SS: Soluble sugars
SOD: Superoxide dismutase
SUS: Sucrose synthase
Swiss-Prot: Swiss-Prot protein database
TPM: Transcripts Per Kilobase of exon model per Million mapped reads
TFs: Transcription factors
*TPS*: Trehalose-phosphate synthase
WGCNA: Weighted correlation network analysis

## References

[1] Anderegg WRL, Kane JM, Anderegg LDL (2013). Consequences of widespread tree mortality triggered by drought and temperature stress. Nat Clim Chang.

[2] Lei P, Liu Z, Li JX, Jin GZ, Xu LP, Ji XM, Zhao XY, Tao L, Meng FJ (2022). Integration of the Physiology, Transcriptome and Proteome Reveals the Molecular Mechanism of Drought Tolerance in Cupressus gigantea. Forests.

[3] Gupta A, Rico-Medina A, Caño-Delgado AI (2020). The physiology of plant responses to drought. Science (New York, NY)..

[4] Fàbregas N, Lozano-Elena F, Blasco-Escámez D, Tohge T, Martínez-Andújar C, Albacete A, Osorio S, Bustamante M, Riechmann JL, Nomura T, Yokota T, Conesa A, Alfocea F, Pérez AF, Fernie AR, Caño-Delgado AI (2018). Overexpression of the vascular brassinosteroid receptor
* BRL3
* confers drought resistance without penalizing plant growth. Nature Commun.

[5] Jia SJ, Li HW, Jiang YP, Tang YL, Zhao GQ, Zhang YL, Yang SJ, Qiu HS, Wang Y, Guo J (2020). Transcriptomic analysis of female panicles reveals gene expression responses to drought stress in maize (Zea mays L). Agronomy..

[6] Costa V, Angelini C, De Feis I, Ciccodicola A (2010). Uncovering the complexity of transcriptomes with RNA-Seq. Biomed Res Int.

[7] Kumar V, Hainaut M, Delhomme N, Mannapperuma C, Immerzeel P, Street NR, Henrissat B, Mellerowicz EJ (2019). Poplar carbohydrate-active enzymes: whole-genome annotation and functional analyses based on RNA expression data. Plant J.

[8] Wei W, Liang DW, Bian XH, Shen M, Xiao JH, Zhang WK, Ma B, Lin Q, Lv J, Chen X (2019). GmWRKY54 improves drought tolerance through activating genes in abscisic acid and Ca2+ signaling pathways in transgenic soybean. Plant J.

[9] Danilevskaya ON, Yu G, Meng X, Xu J, Stephenson E, Estrada S, Chilakamarri S, Zastrow-Hayes G, Thatcher S (2019). Developmental and transcriptional responses of maize to drought stress under field conditions. Plant Direct.

[10] Wang F (2021). Seed propagation techniques of Gleditsia sinensis seedlings. Forest By-Product Speciality China.

[11] Liu FH, Wang XR, Zhao Y, He KQ (2022). Effects of different temperatures on growth and physiological characteristics of g sinensis seedlings. J Mountain Agriculture Biology..

[12] Qin KY (2020). The research of response of seed germination and seedling growth of Gleditsia microphylla to drought stress and exogenous Ca^2+^.

[13] Liu Q, Yang J, Wang XR, Zhao Y (2023). Studies on Pollen Morphology, pollen vitality and preservation methods of Gleditsia sinensis Lam (Fabaceae). Forests..

[14] Tian HH, Yang J, Lu CY, Xiao F, Zhao Y (2022). Phenotypic diversity analysis of province of natural g sinensiss in Guizhou. Acta Botanica Boreali-Occidentalia Sinica..

[15] Xiao F, Zhao Y, Wang XR, Jian XY (2023). Differences in the Growth of Seedlings and the Selection of Fast-Growing Species in the Gleditsia Genus. Forests.

[16] Zhang YY (2020). Compensatory effects of Rewatering after Droughton Growth and Physiology of Platycladus orientalis.

[17] Chen S, Zhou Y, Chen Y, Gu J (2018). fastp: an ultra-fast all-in-one FASTQ preprocessor. Bioinformatics.

[18] Grabherr MG, Haas BJ, Yassour M, Levin JZ, Thompson DA, Amit I, Adiconis X, Fan L, Raychowdhury R, Zeng Q (2011). Full-length transcriptome assembly from RNA-Seq data without a reference genome. Nat Biotechnol.

[19] Li WZ, Godzik A (2006). Cd-hit: a fast program for clustering and comparing large sets of protein or nucleotide sequences. Bioinformatics.

[20] Liu CG, Duan N, Chen XN, Li HQ, Zhao XL, Duo PZ, Wang J, Li QK. Metabolic Pathways Involved in the Drought Stress Response of Nitraria tangutorum as Revealed by Transcriptome Analysis. Forests. 2022;13(4):509.

[21] Langmead B, Salzberg S (2012). Fast gapped-read alignment with Bowtie 2. Nat Meth.

[22] Li B, Dewey CN (2011). RSEM: accurate transcript quantification from RNA-Seq data with or without a reference genome. BMC Bioinformatics.

[23] Love MI, Huber W, Anders S (2014). Moderated estimation of fold change and dispersion for RNA-seq data with DESeq2. Genome Biol.

[24] Langfelder P, Horvath S (2008). WGCNA: an R package for weighted correlation network analysis. BMC Bioinformatics.

[25] Yu  GC, Wang  LG, Han  YY, He  QY (2012). clusterProfiler an R package for comparing biological themes among gene clusters Omics. J Integrative Biology.

[26] Blighe K, Lun A. PCAtools: Everything Principal Components Analysis. R package version. 2021;2(0).

[27] Xiao F, Zhao Y, Wang X, Jian X (2023). Full-length transcriptome characterization and comparative analysis of G sinensis. BMC genomics..

[28] Yang XY, Zhao TY, Rao P, Gao K, Yang X, Chen Z, An XM (2019). Transcriptome profiling of Populus tomentosa under cold stress. Ind Crops Prod.

[29] R Core Team. R: A language and environment for statistical computing team RDCVienna, Austria 2006. 2013:275-86.

[30] Wang J, Sun L, Zhang HW, Jiao B, Wang HB, Zhou S (2023). Transcriptome analysis during vernalization in wheat (Triticum aestivum L). BMC Genomic Data..

[31] Begara-Morales JC, Sánchez-Calvo B, Chaki M, Valderrama R, Mata-Pérez C, López-Jaramillo J, Padilla MN, Carreras A, Corpas FJ, Barroso JB (2014). Dual regulation of cytosolic ascorbate peroxidase (APX) by tyrosine nitration and S-nitrosylation. J Exp Bot.

[32] Guerzoni JTS, Belintani NG, Moreira RMP, Hoshino AA, Domingues DS, Filho JCB, Vieira LG. Stress-induced Δ1-pyrroline-5-carboxylate synthetase (P5CS) gene confers tolerance to salt stress in transgenic sugarcane. Acta Physiologiae Plantarum. 2014;36:2309–19.

[33] Yamchi A, Rastgar Jazii F, Mousavi A, Karkhane A. Proline accumulation in transgenic tobacco as a result of expression of Arabidopsis Δ 1-pyrroline-5-carboxylate synthetase (P5CS) during osmotic stress. J Plant Biochem Biotechnol. 2007;16:9–15.

[34] Zhang SY, Liu YC, Jia SQ, Li YT, Huang YR, Yang MS, Zhang J. Physiological response and transcriptome analysis of potted seedlings Ulmus pumila “Zhonghua Jinye” under drought stress. J Hebei Agricultural University. 2022;45(01):69–78.

[35] Ma W, Zou LY, Zhao HX, Ge FW (2022). Analysis of the Main Metabolic Pathway of Drought Resistance of Dontostemon elegans based on Transcriptome Sequencing. Molecular Plant Breeding.

[36] Zhao L, Wang J, Wang F. Transcriptome Analysis of Potato Stem under Drought Stress Simulated by PEG. Acta Botan Boreali-Occiden Sin. 2020;40(3):403–12.

[37] Zhao NN, Cui SL, Li XK, Liu BK, Deng HT, Liu YR, Hou MY, Yang XL, Mu GJ, Liu LF (2021). Transcriptome and co-expression network analyses reveal differential gene expression and pathways in response to severe drought stress in peanut (Arachis hypogaea L). Frontiers in Genetics..

[38] Wang ZH, Wei YQ, Zhao YR, Wang YJ. A transcriptomic study of physiological responses to drought and salt stress in sweet sorghum seedlings. Acta Pratacul Sin. 2022;31(3):71–84.

[39] Lin Z, Li Y, Wang YB, Liu XL, Ma L, Zhang ZG, Mu C, Zhang Y, Peng L, Xie SJ (2021). Initiation and amplification of SnRK2 activation in abscisic acid signaling. Nat Commun.

[40] Fang YJ, Xiong LZ (2015). General mechanisms of drought response and their application in drought resistance improvement in plants. Cell Mol Life Sci.

[41] Peng YY, Yan HH, Guo LC, Ren CZ. Evaluation and selection drought-resistance of germplasm resources of Avena species with different types of ploidy. Acta Ecol Sin. 2011;31(9):2478–91.

[42] Liu B, Liang CJ (2005). Recent Advance of Catalase in Organism. Chinese Agricultural Science Bulletin..

[43] Zuo KF. Identification of drought-resistant germplasms in Brassica napus L. and analysis of related genes. Xianyang: Northwest Agriculture and Forestry University; 2020.

[44] Sun XC, Li HR (2022). Metabolomics Analysis of Platycodon grandiflorus Leaves Under Drought Stress. Northern Horticulture.

[45] Shinozaki K, Yamaguchi-Shinozaki K, Seki M (2003). Regulatory network of gene expression in the drought and cold stress responses. Curr Opin Plant Biol.

[46] Li YJ, Zhu DH, Dong DK. Progress in the study of transcription factors related to drought resistance in soybean. J Yangtze University (Natural Science Edition). 2014;11(5):50–6.

[47] Yang JT, Yu Y, Gao XR, Pi RX (2021). Advances in Plant MYB Transcription Factors Regulation Mechanisms to Various Stress. J Hangzhou Normal University (Natural Science Edition)..

[48] Li J, Chen LL, Chang Y (2021). Cloning of MYB2 gene from Dryopteris fragrans and its response to ABA and drought stress. J Northeast Agric University.

[49] Li XY, Zhou JW, Yan ZY, Chen X (2020). Sequencing and analysis of the transcriptome to reveal regulation of gene expression in Salvia miltiorrhiza under moderate drought stress. Chinese Traditional and Herbal Drugs.

[50] Wang WY, Xu MY, Wang Guo P, Galili G. New insights into the metabolism of aspartate-family amino acids in plant seeds. Plant reproduction. 2018;31(3):203-11. 10.1007/s00497-018-0322-9 29399717

[51] Guo RR (2015). The Establishment of Grape Somatic Embryo Regeneration System and the Functional Study of Aspartic Proteases Family Gene in Grape.

[52] Qu LN, Sun MS, Li XM, He RQ, Zhong M, Luo D, Liu XM, Zhao XY (2020). The Arabidopsis F-box protein FOF2 regulates ABA-mediated seed germination and drought tolerance. Plant Sci.

[53] Wang S, Yuan K, He QG, Hu YY, Feng CT, Wang ZH, Liu JP, Liu H (2022). Cloning and Expression Analysis of HbCXXS1, a Thioredoxin Gene in Hevea brasiliensis. Biotechnology Bulletin..

[54] Xia DX, Guan QJ, Jin SM, Li YJ, Lang H, Zhang XX, et al. The Relationship of Arabidopsis thaliana Thioredoxin M1-type 1 (AtTRX m1) Gene with Environmental Stress. Molecular Plant Breeding. 2007;(1):21–26.

[55] Skaar JR, Pagan JK, Pagano M (2013). Mechanisms and function of substrate recruitment by F-box proteins. Nat Rev Mol Cell Biol.

[56] Song AP, Zhu XR, Chen FD, Gao HS, Jaifu J, Chen S (2014). A chrysanthemum heat shock protein confers tolerance to abiotic stress. Int J Molecular Sci.

[57] Li T, Chen BD. Arbuscular mycorrhizal fungi improve drought tolerance of maize plants by up-regulating of aquaporin gene expression in roots and the fungi themselves. Chinese J Plant Ecology. 2012;36(9):973–81.

