# Supplementary material for: Transcriptome analysis reveals regulatory mechanisms of different drought-tolerant Gleditsia sinensis seedlings under drought stress
Source: BMC Genom Data. 2024 Mar 13;25:29. doi: 10.1186/s12863-024-01216-y (PMC10935782; doi:10.1186/s12863-024-01216-y)
Supplement: Supplementary file 1 — Supplementary Material 1. [file 12863_2024_1216_MOESM1_ESM.docx]

**Supplementary Information**

**Additional file 1: Table S1.** Sample RNA quality test results.

| Name | Concentration  (ng/ul) | OD260/280 | OD260/230 | RQN |
| --- | --- | --- | --- | --- |
| CHS 1 | 1243.4 | 2.15 | 2.29 | 6.5 |
| CHS 2 | 930.5 | 2.16 | 2.16 | 4.6 |
| CHS 3 | 978.8 | 2.16 | 2.29 | 5.9 |
| DHS 1 | 1105.1 | 2.17 | 2.35 | 7.8 |
| DHS 2 | 1029.2 | 2.17 | 2.21 | 8.0 |
| DHS 3 | 1155.9 | 2.17 | 2.27 | 7.6 |
| SHS 1 | 1046 | 2.16 | 2.26 | 8.3 |
| SHS 2 | 817.2 | 2.17 | 1.94 | 7.4 |
| SHS 3 | 884.4 | 2.14 | 2.27 | 8.5 |
| rHS 1 | 956.8 | 2.18 | 2.18 | 7.8 |
| rHS 2 | 940.7 | 2.18 | 1.91 | 7.4 |
| rHS 3 | 961.3 | 2.17 | 2.28 | 7.7 |
| RHS 1 | 919.6 | 2.19 | 2.3 | 7.6 |
| RHS 2 | 824.4 | 2.18 | 2.13 | 7.8 |
| RHS 3 | 691.6 | 2.18 | 2.12 | 7.9 |
| CHR 1 | 957.8 | 2.12 | 2.29 | 6.6 |
| CHR 2 | 571.9 | 2.13 | 2.23 | 6.0 |
| CHR 3 | 746.5 | 2.17 | 2.22 | 6.8 |
| DHR 1 | 1166.3 | 2.15 | 2.26 | 6.4 |
| DHR 2 | 1308.7 | 2.17 | 1.9 | 7.2 |
| DHR 3 | 958.9 | 2.18 | 2.2 | 6.9 |
| SHR 1 | 1146.5 | 2.18 | 2.04 | 7 |
| SHR 2 | 1400.4 | 2.16 | 2.19 | 7 |
| SHR 3 | 1178.6 | 2.16 | 2.34 | 6.9 |
| rHR 1 | 1085.5 | 2.08 | 2.31 | 7.1 |
| rHR 2 | 1018.9 | 2 | 2.27 | 7.8 |
| rHR 3 | 981.8 | 2.09 | 2.31 | 7.2 |
| RHR 1 | 937.6 | 2.17 | 2.3 | 6.3 |
| RHR 2 | 1225.6 | 2.18 | 2.18 | 5.7 |
| RHR 3 | 1192.5 | 2.17 | 2.28 | 6.6 |


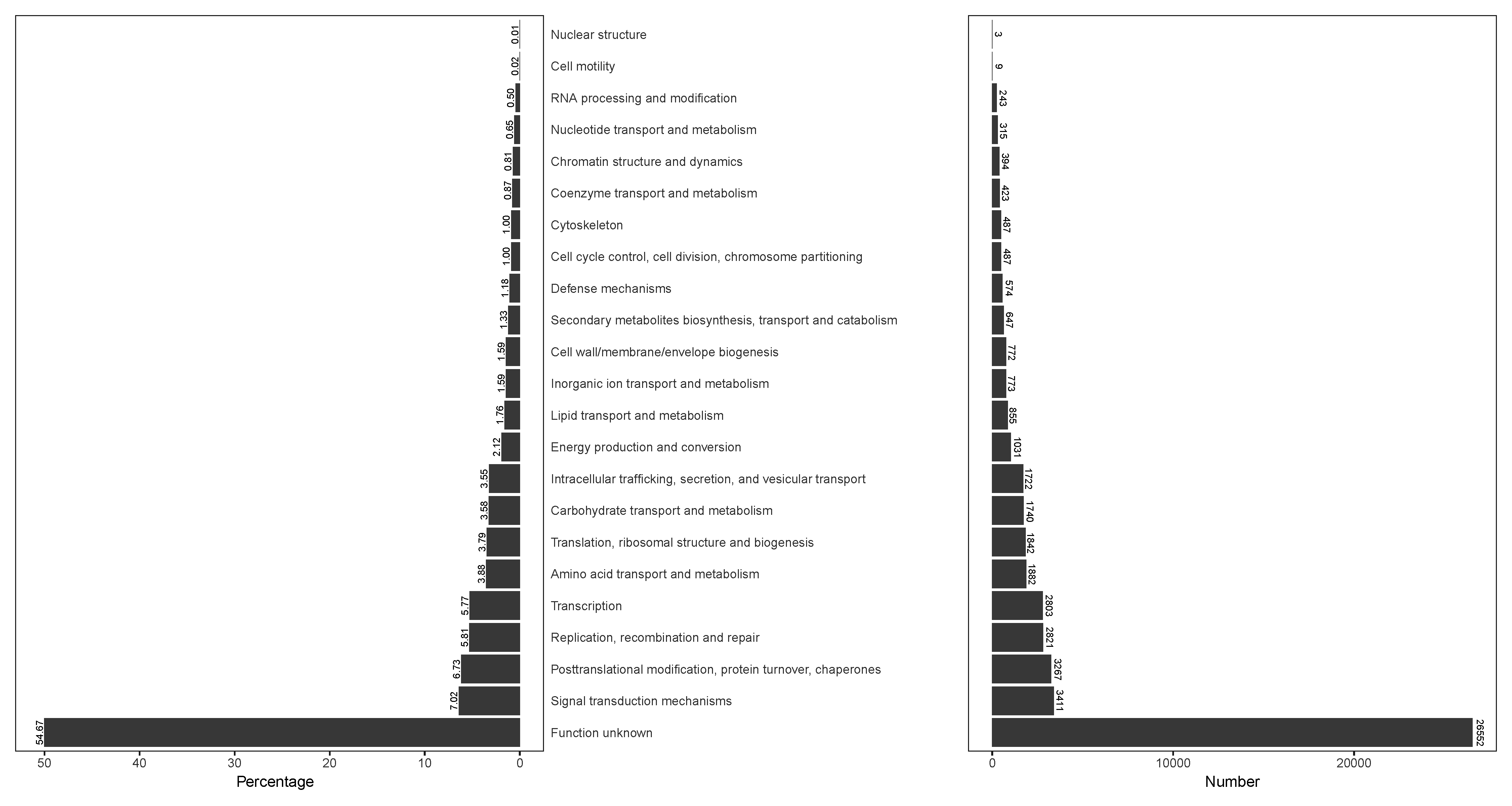


**Additional file 2: Figure S1** Functional classification map of COG.


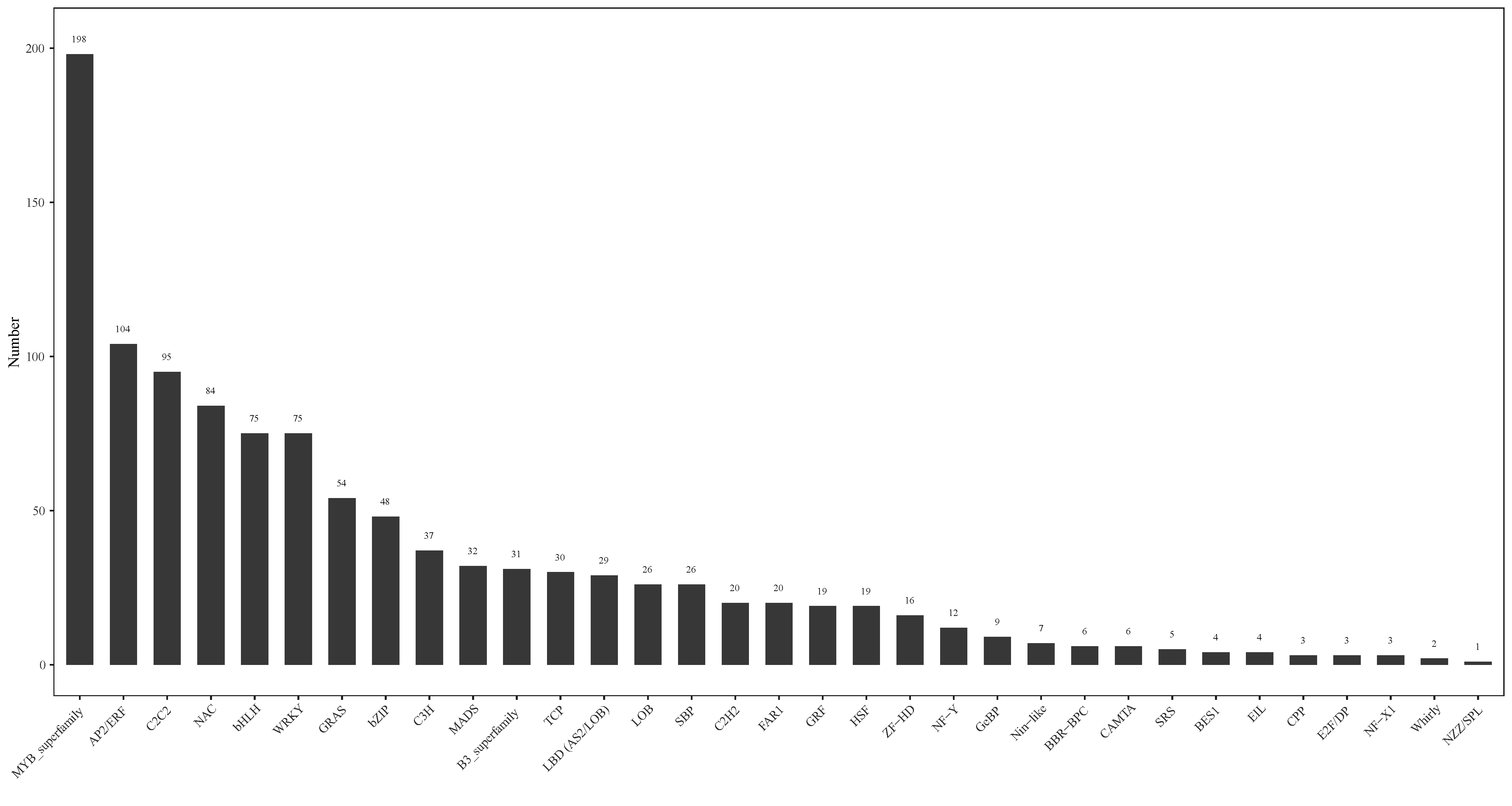


**Additional file 3: Figure S2** Classification chart of transcription factor family.


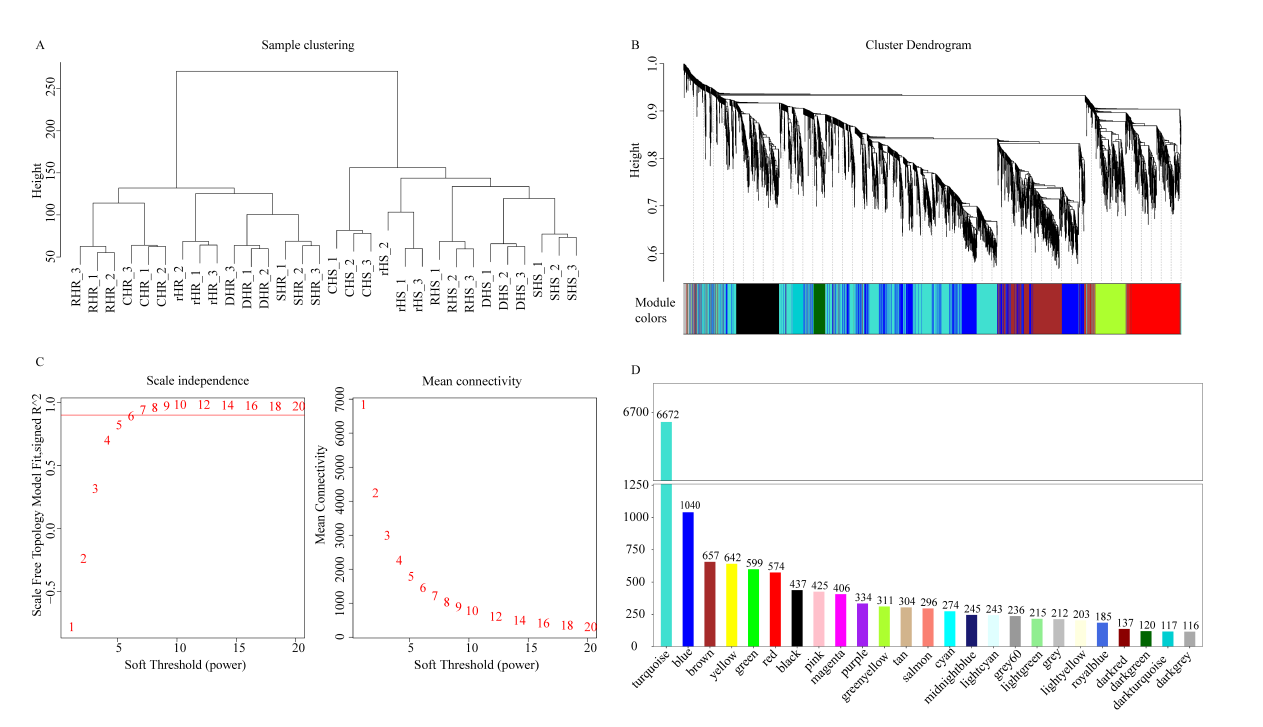


**Additional file 4: Figure S3** Co-expression network module analysis.

Note: A: *G. sinensis* transcriptome sample expression hierarchical clustering plot; B: *G. sinensis* transcriptome clustering and module identification; C: The determination of soft threshold; D: Distribution of gene numbers in co-expression modules.


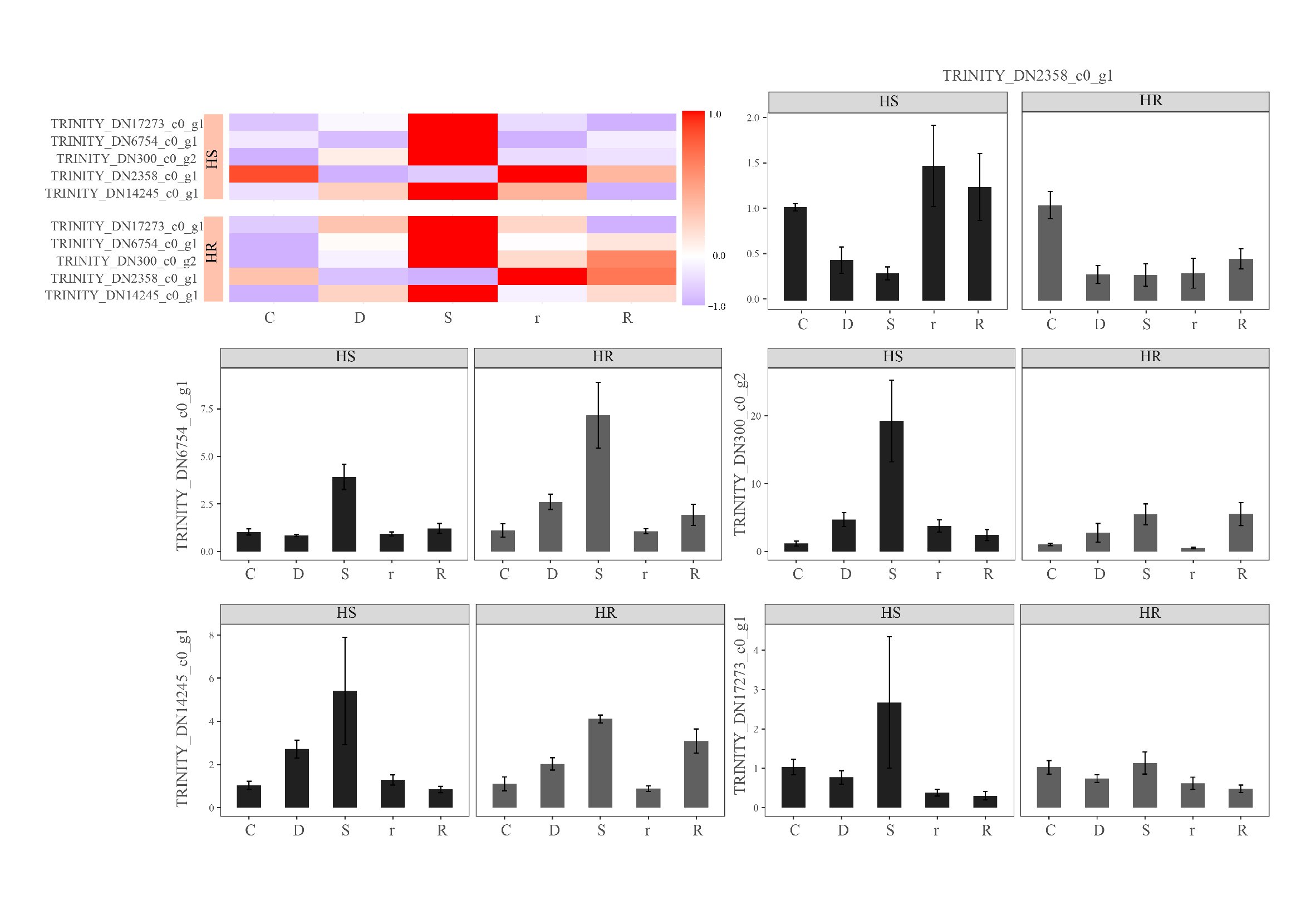


**Additional file 5: Figure S4** Comparison of qRT-PCR and RNA-Seq.


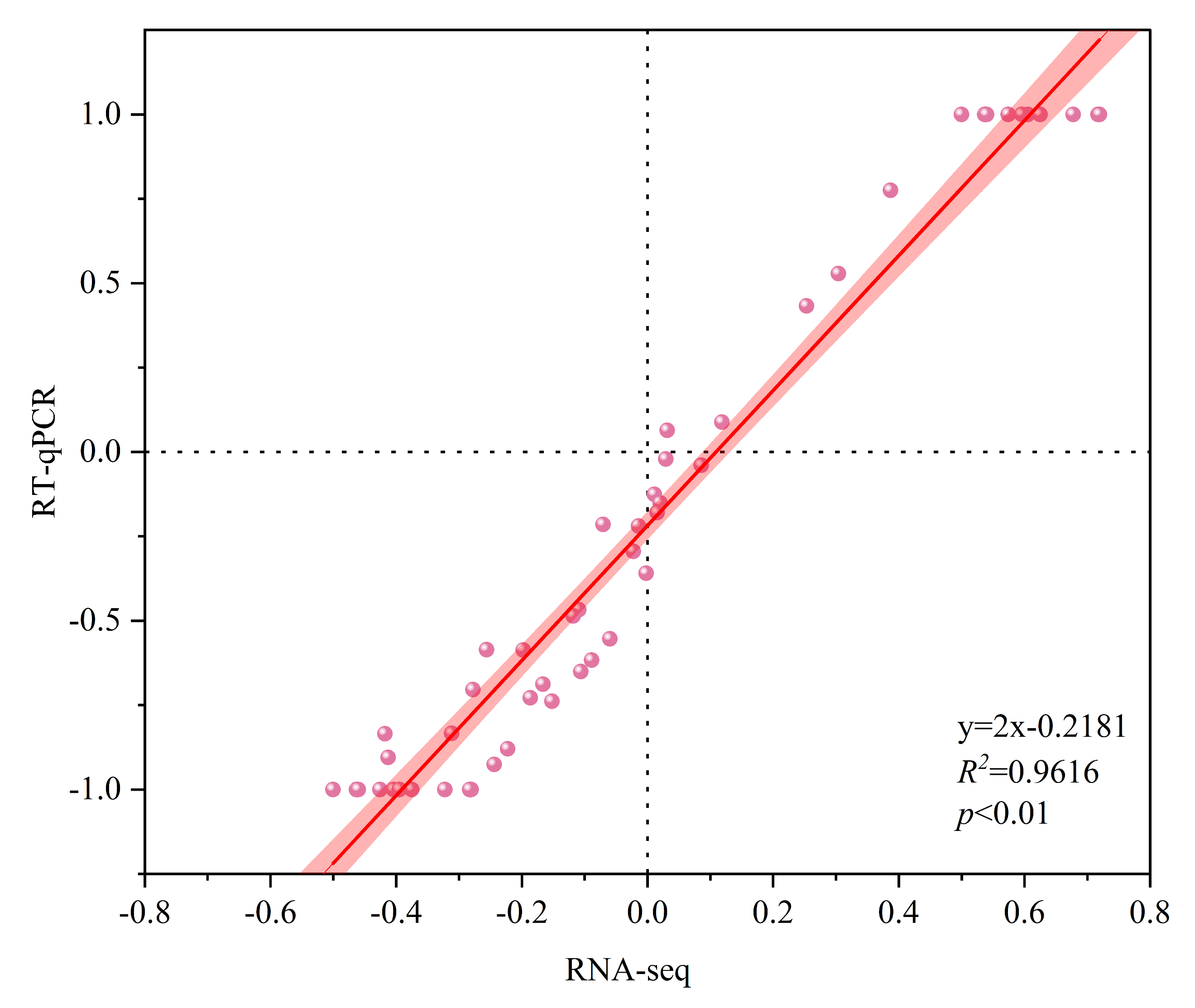


**Additional file 6: Figure S5** Correlation between the relative expression of transcriptome and RT-qPCR.


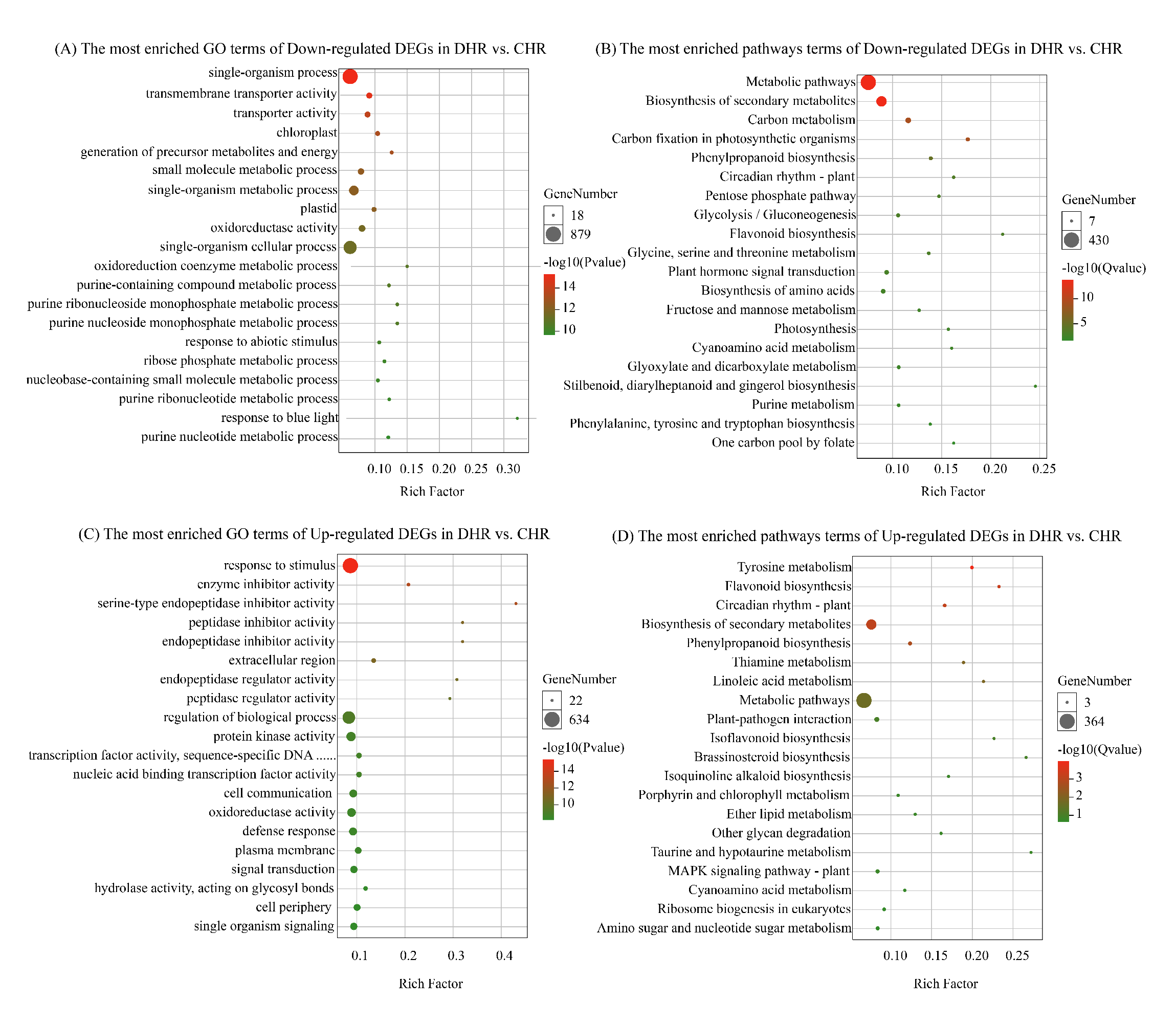


**Additional file 7: Figure S6** GO and KEGG enrichment analysis of DEGs in *G. sinensis* HR families in response to mild drought stress.


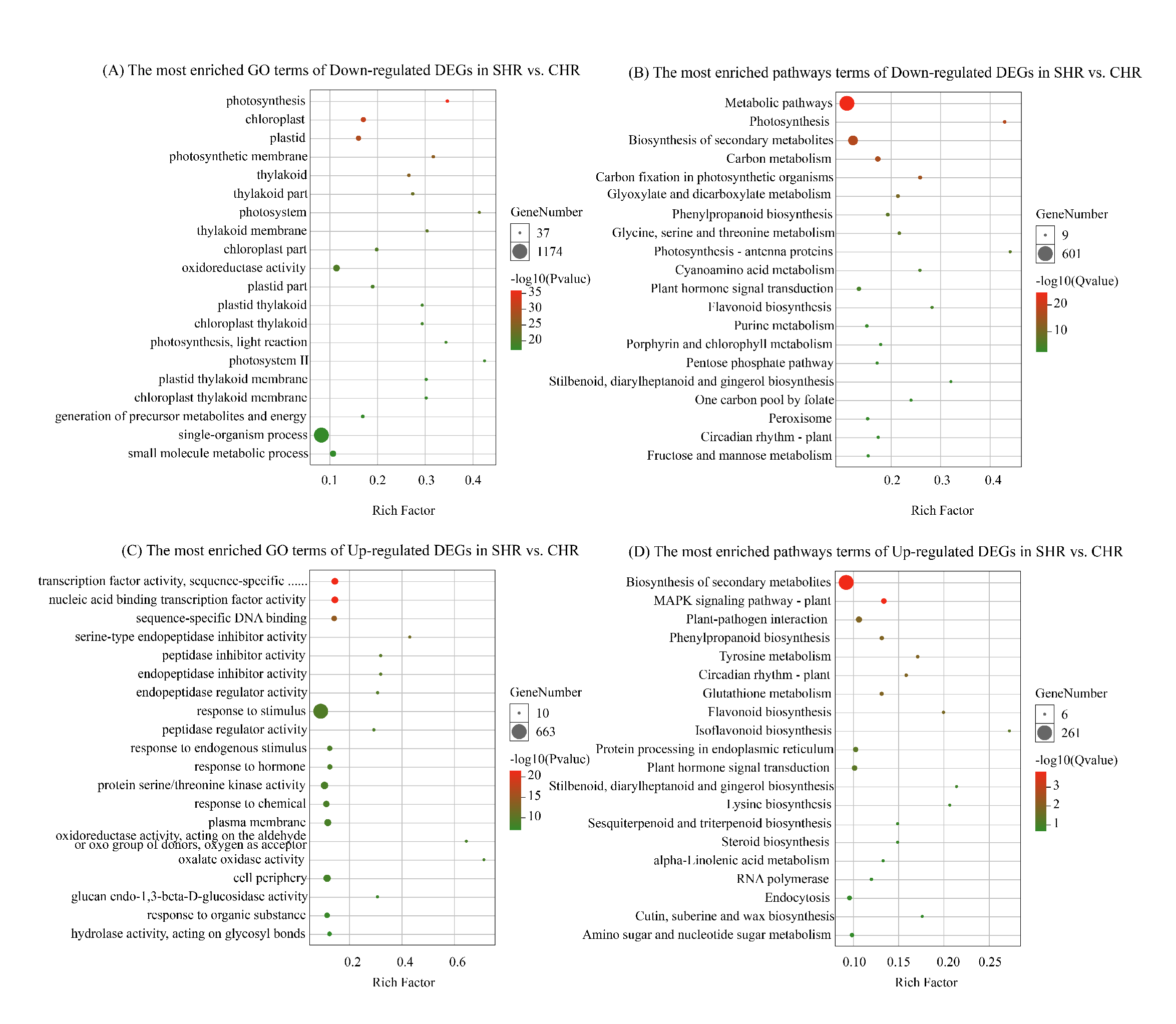


**Additional file 8: Figure S7** GO and KEGG enrichment analysis of DEGs in *G. sinensis* HR family response to severe drought stress.


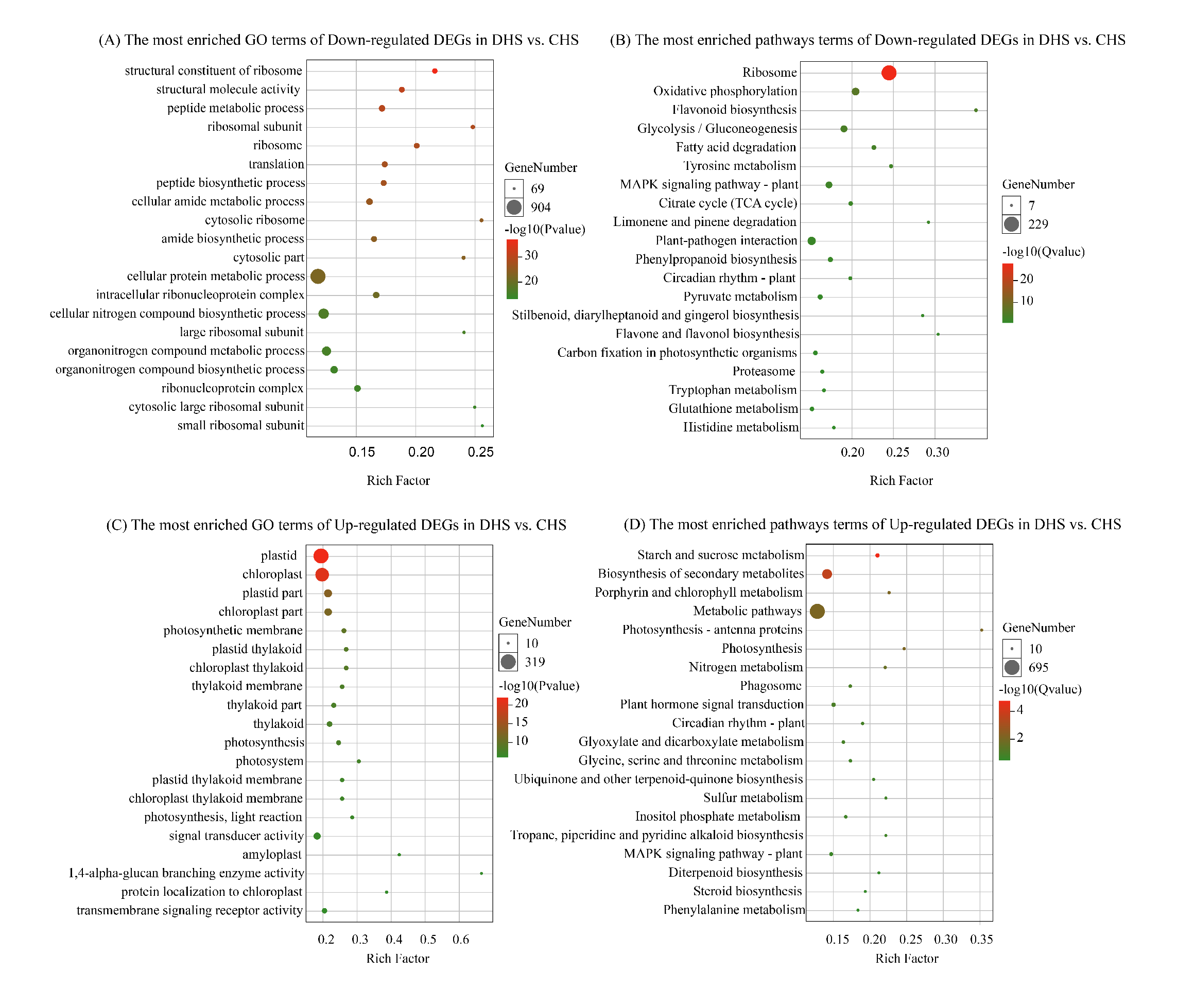


**Additional file 9: Figure S8** GO and KEGG enrichment analysis of DEGs in *G. sinensis* HS families in response to mild drought stress.


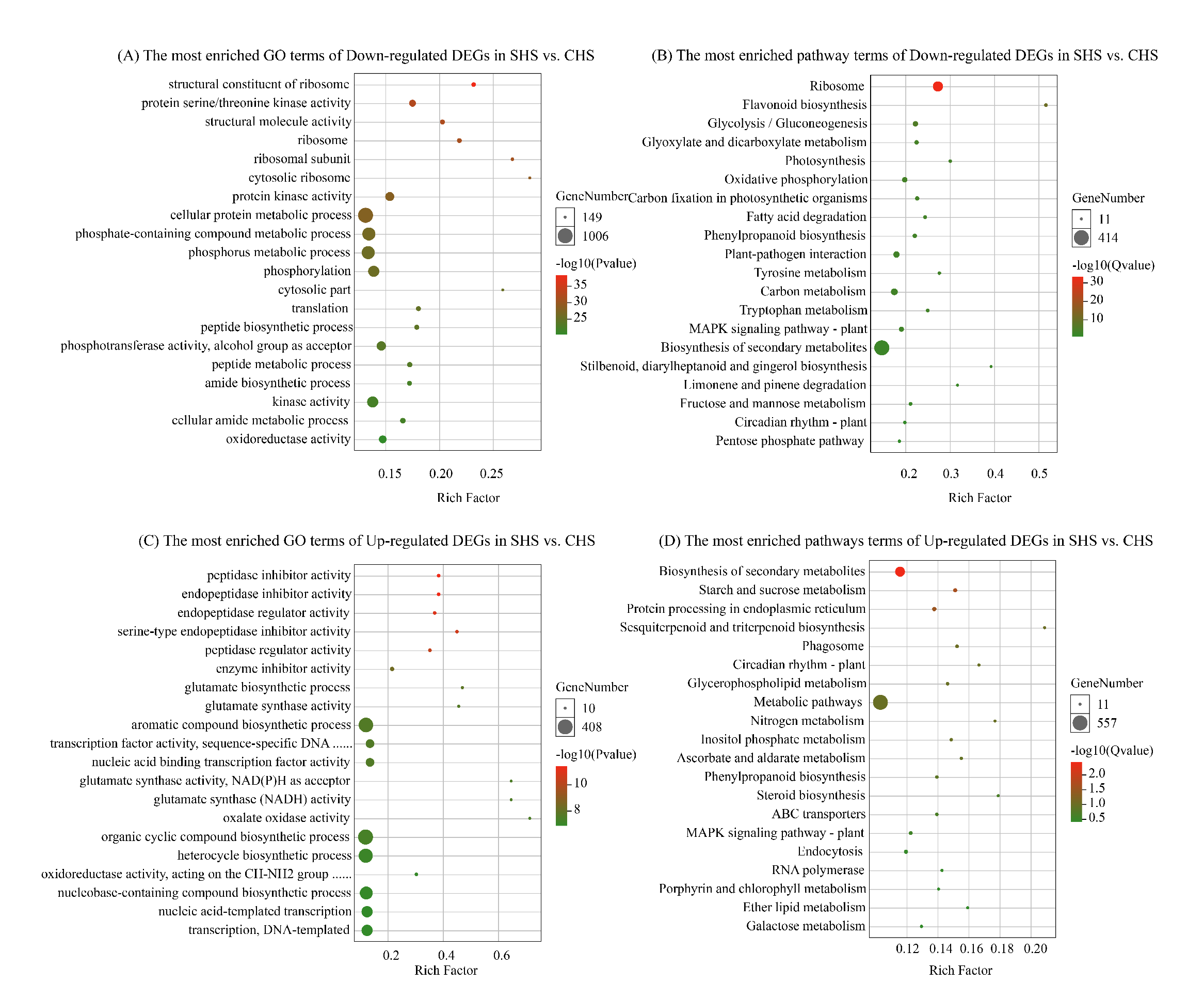


**Additional file 10: Figure S9** GO and KEGG enrichment analysis of DEGs in response to mild drought stress in *G. sinensis* HS families.


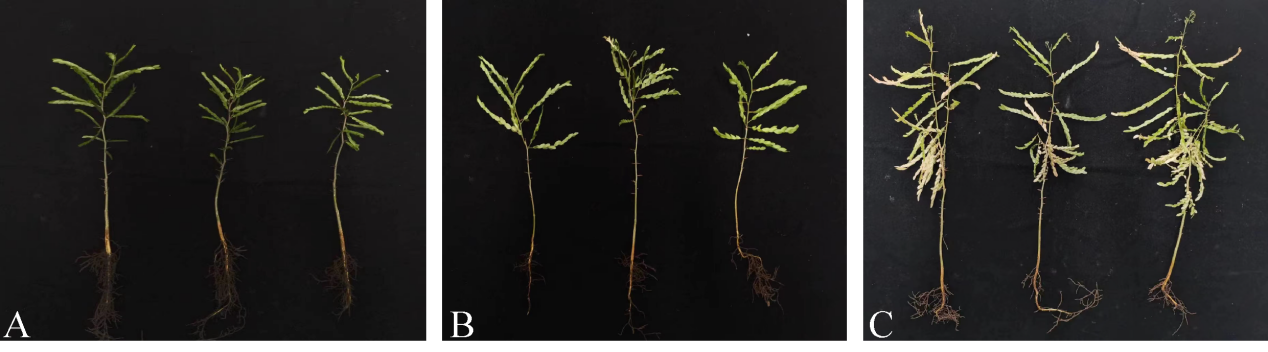


**Additional file 11: Figure S10** Morphological pictures of *G. sinensis seedlings* with different drought levels.

Note: A, control; B, mild drought; C, severe drought.
